# Supplementary material for: Protein-encapsulated doxorubicin reduces cardiotoxicity in hiPSC-cardiomyocytes and cardiac spheroids while maintaining anticancer efficacy
Source: Stem Cell Reports. 2023 Aug 31;18(10):1913–24. doi: 10.1016/j.stemcr.2023.08.005 (PMC10656302; doi:10.1016/j.stemcr.2023.08.005)
Supplement: Document S1. Figures S1–S4 and supplemental experimental procedures [file mmc1.pdf]

Stem Cell Reports, Volume *18*

Supplemental Information

Protein-encapsulated doxorubicin reduces cardiotoxicity in hiPSC-cardiomyocytes and cardiac spheroids while maintaining anticancer efficacy

Madelyn Arzt, Bowen Gao, Maedeh Mozneb, Stephany Pohlman, Romina B. Cejas, Qizhi Liu, Faqing Huang, Changjun Yu, Yi Zhang, Xuemo Fan, Amelia Jenkins, Armando E. Giuliano, Paul W. Burridge, Xiaojiang Cui, and Arun Sharma

SUPPLEMENTAL FIGURES AND LEGENDS

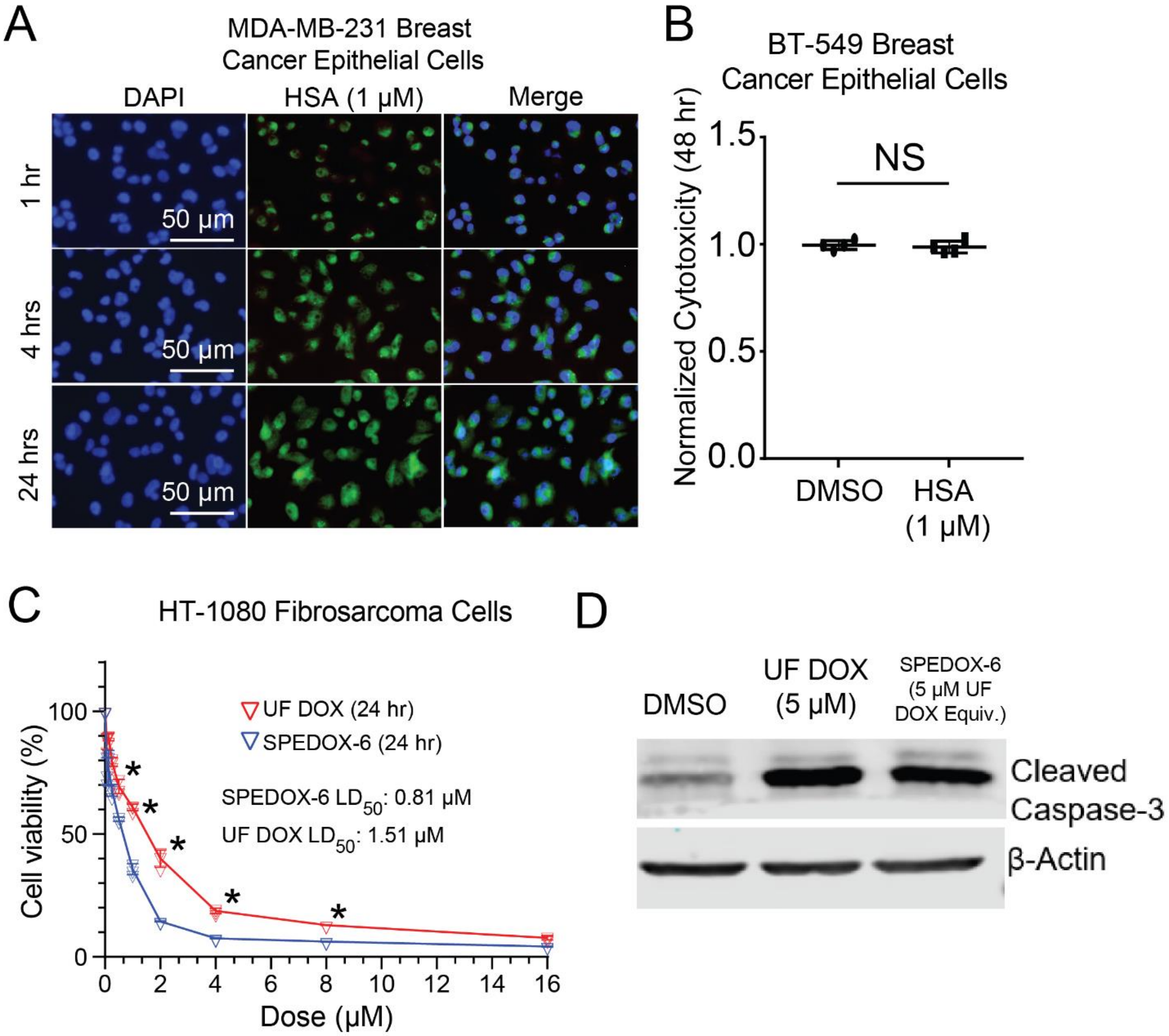

Figure S1 (related to Figure 1): Evaluation of SPEDOX-6 cytotoxicity in multiple human cancer cell lines. (A) Immunofluorescence staining following treatment of MDA-MB-231 human breast cancer cells with human serum albumin (HSA). HSA exhibits rapid nuclear accumulation in MDA-MB-231 cells within hours of treatment. **(B)** BT-549 breast cancer epithelial cells treated with either DMSO or HSA for 48 hours show no significant (NS) difference in cytotoxicity. * indicates $p<0.05$ by Student's t test. NS indicates non-significance. N=4 independent experiments. **(C)** Cell viability dose response assay conducted with unformulated doxorubicin (UF DOX) and SPEDOX-6 on HT-1080 fibrosarcoma cells for 24 hours. * indicates $p<0.05$ by Student's t test. N=3 independent experiments. **(D)** Western blot assay examining the protein expression of cleaved caspase-3 (apoptosis marker) in HT-1080 fibrosarcoma cells after UF DOX or SPEDOX-6 treatment.

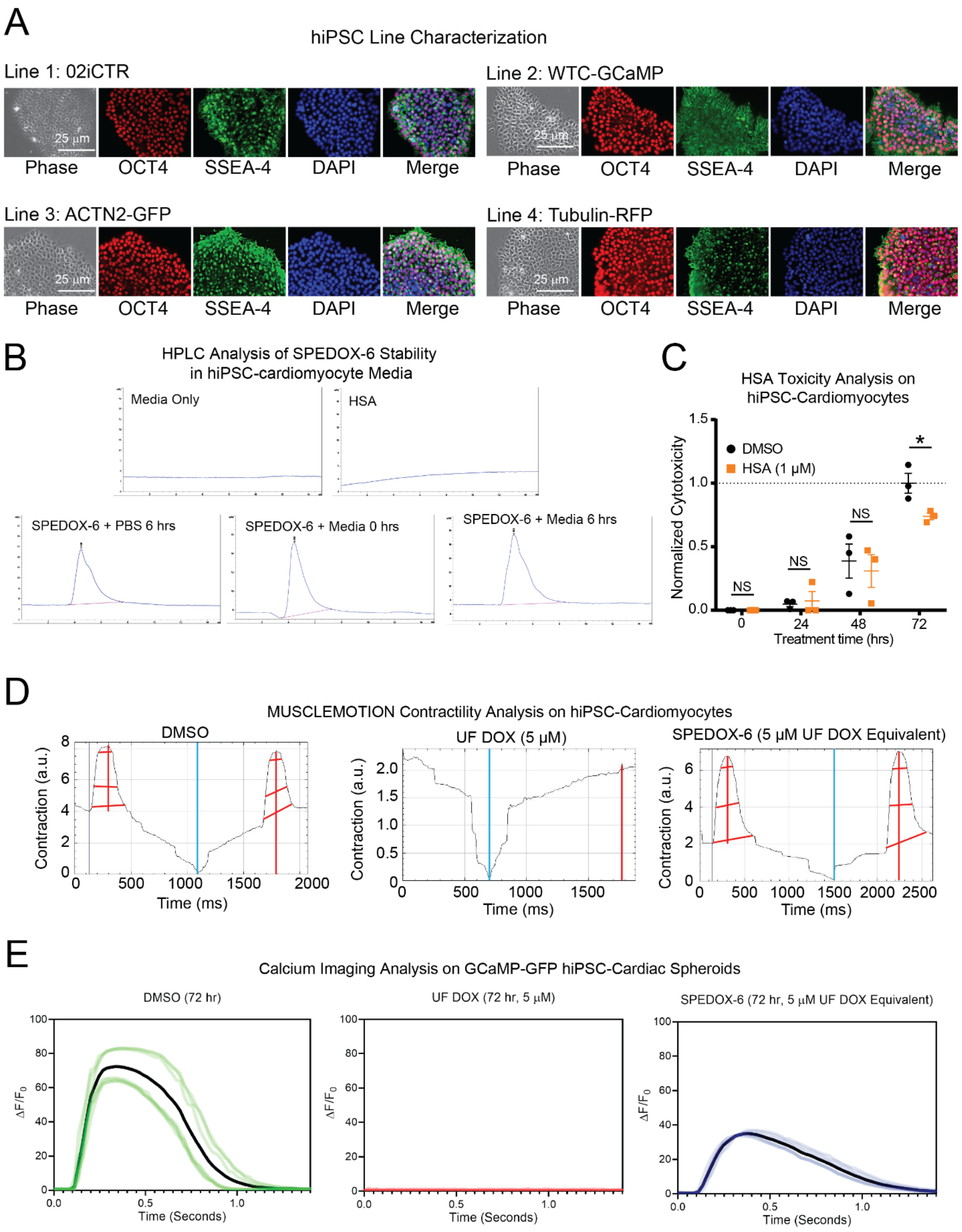
A
hiPSC Line Characterization
Line 1: 02iCTR
25 µm
Phase
OCT4
SSEA-4
DAPI
Merge
Line 2: WTC-GCaMP
Line 3: ACTN2-GFP
Line 4: Tubulin-RFP
B
HPLC Analysis of SPEDOX-6 Stability in hiPSC-cardiomyocyte Media
Media Only
HSA
SPEDOX-6 + PBS 6 hrs
SPEDOX-6 + Media 0 hrs
SPEDOX-6 + Media 6 hrs
C
HSA Toxicity Analysis on hiPSC-Cardiomyocytes
DMSO
HSA (1 µM)
Normalized Cytotoxicity
NS
*
Treatment time (hrs)
D
MUSCLEMOTION Contractility Analysis on hiPSC-Cardiomyocytes
DMSO
UF DOX (5 µM)
SPEDOX-6 (5 µM UF DOX Equivalent)
Contraction (a.u.)
Time (ms)
E
Calcium Imaging Analysis on GCaMP-GFP hiPSC-Cardiac Spheroids
DMSO (72 hr)
UF DOX (72 hr, 5 µM)
SPEDOX-6 (72 hr, 5 µM UF DOX Equivalent)
ΔF/F0
Time (Seconds)

Figure S2 (related to Figure 2, 3, 4): Characterization of hiPSC morphology and subsequent evaluation of differentiated hiPSC-cardiomyocyte HSA uptake and SPEDOX-6-induced toxicity (A) Immunofluorescence staining of hiPSC lines used in this project for protein markers of pluripotent stem cells, including OCT4 and SSEA-4. These markers indicate a high level of pluripotency gene expression in undifferentiated hiPSCs, a reflection of their overall health as pluripotent stem cells. These hiPSCs are then used for differentiations into hiPSC-derived cardiomyocytes (hiPSC-CMs), cardiac fibroblasts (hiPSC-CFs), and endothelial cells (hiPSC-ECs). **(B)** High performance liquid chromatography reveals SPEDOX-6 stability in cardiomyocyte culture medium. High performance liquid chromatography (HPLC) using a size exclusion Protein-Pak 125 Column (Waters, MAQ, 7.8 mm x 300 mm) column. The analytes were monitored at 480 nm. Large molecules, such as proteins, were eluted first, whereas small molecules were retained for a more extended period of time before elution. Chromatography results with media only, human serum albumin (HSA), SPEDOX-6, SPEDOX-6 added to media for 0 hours, or SPEDOX-6 added to media for 6 hours. Results indicate that 6 hours of mixing cardiomyocyte cell culture medium with SPEDOX-6 did not alter the absorption profile, suggesting that SPEDOX-6 stability is not substantially affected by the culture medium. Y-axis: absorption at 480 nm, maximum absorption wavelength of doxorubicin. **(C)** Human serum albumin (HSA) treatment alone in hiPSC-cardiomyocytes (hiPSC-CMs) does not enhance cytotoxicity above DMSO. hiPSC-CMs treated with HSA show no enhancement in cytotoxicity above DMSO via lactate dehydrogenase (LDH) assay. N=3 independent experiments. * indicates $p<0.05$ by Student's t test. **(D)** Automated analysis of 2D hiPSC-CM contractility after drug treatment using MUSCLEMOTION software shows improvement in contractility in SPEDOX-6 treatment compared to unformulated doxorubicin (UF DOX). Videos of hiPSC-cardiomyocyte (hiPSC-CM) contractility after (A) DMSO, (B) UF DOX, or (C) SPEDOX-6 treatment were analyzed using MUSCLEMOTION open-source software. This tool is a plugin for ImageJ that enables automated measurement of cardiomyocyte movement from input video files. Blue lines indicate the reference frame used for cell displacement measurements (Y-axis) over time (X-axis). Red lines highlight automatically detected cellular contractions. **(E)** Analysis of calcium cycling after drug treatment in hiPSC-cardiac spheroids (hiPSC-CSs) shows improvement in calcium handling in SPEDOX-6 treatment compared to unformulated doxorubicin (UF DOX). Videos were taken of hiPSC-CSs containing WTC-GCaMP calcium reporter hiPSC-CMs after (A) DMSO, (B) UF DOX, or (C) SPEDOX-6 treatment. See Supplemental Video 4.

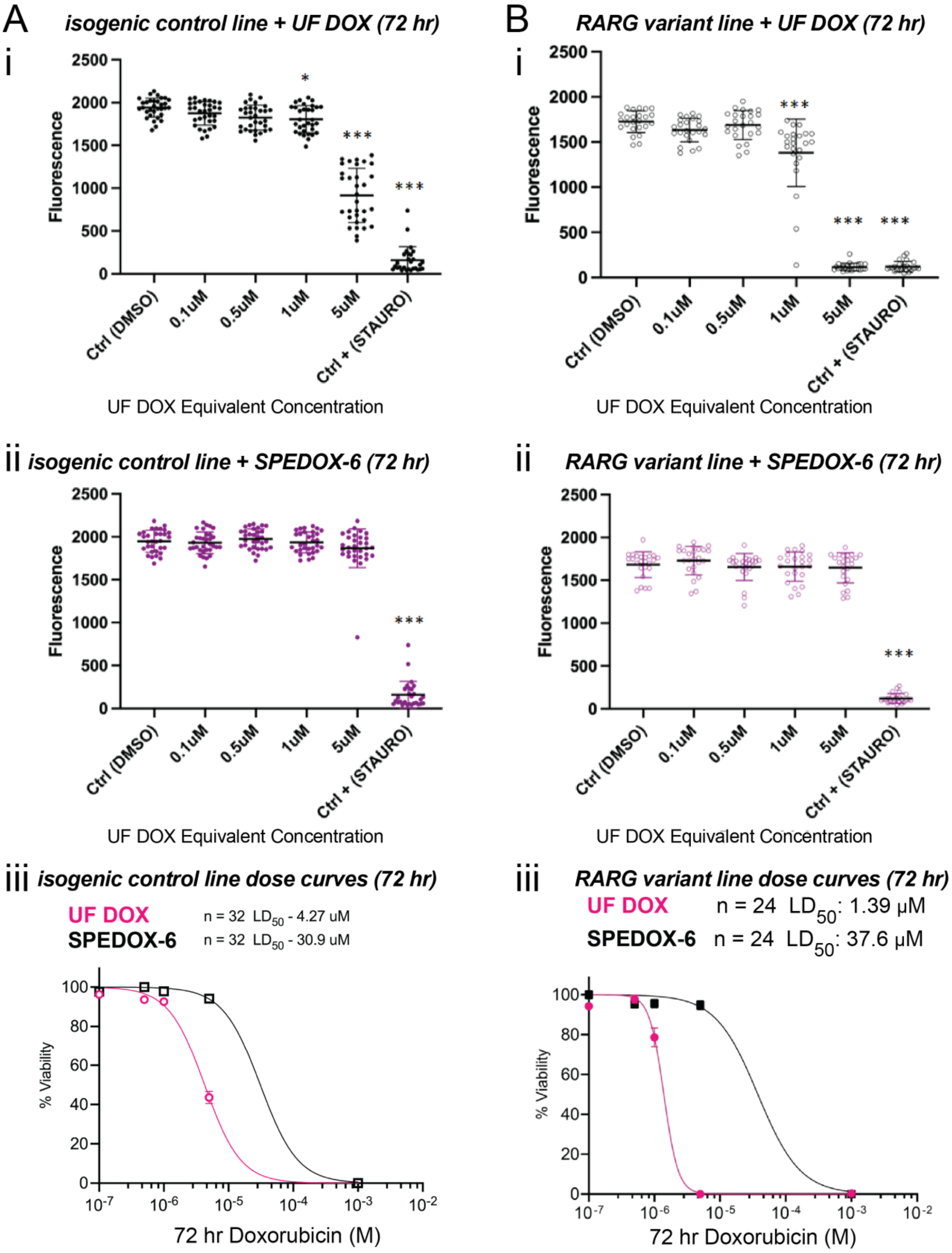
A
isogenic control line + UF DOX (72 hr)
i
2500
2000
1500
1000
500
0
Fluorescence
*
***
***
Ctrl (DMSO)
0.1uM
0.5uM
1uM
5uM
Ctrl + (STAURO)
UF DOX Equivalent Concentration
B
RARG variant line + UF DOX (72 hr)
i
Fluorescence
***
***
***
Ctrl (DMSO)
0.1uM
0.5uM
1uM
5uM
Ctrl + (STAURO)
UF DOX Equivalent Concentration
ii isogenic control line + SPEDOX-6 (72 hr)
Fluorescence
***
Ctrl (DMSO)
0.1uM
0.5uM
1uM
5uM
Ctrl + (STAURO)
UF DOX Equivalent Concentration
ii
RARG variant line + SPEDOX-6 (72 hr)
Fluorescence
***
Ctrl (DMSO)
0.1uM
0.5uM
1uM
5uM
Ctrl + (STAURO)
UF DOX Equivalent Concentration
iii isogenic control line dose curves (72 hr)
UF DOX n = 32 LD50 - 4.27 uM
SPEDOX-6 n = 32 LD50 - 30.9 uM
% Viability
100
80
60
40
20
0
10-7
10-6
10-5
10-4
10-3
10-2
72 hr Doxorubicin (M)
iii
RARG variant line dose curves (72 hr)
UF DOX n = 24 LD50: 1.39 μM
SPEDOX-6 n = 24 LD50: 37.6 μM
% Viability
72 hr Doxorubicin (M)

Figure S3 (related to Figure 2): SPEDOX-6 induces less cytotoxicity than unformulated doxorubicin (UF DOX) in isogenic and patient-specific hiPSC-cardiomyocytes (hiPSC-CMs) harboring single nucleotide polymorphism (rs2229774) in retinoic acid receptor-γ (RARG). Reazurin blue fluorescence-based cell viability dose response assay with UF DOX or SPEDOX-6 treatment for 72 hours in **(A)** an isogenic control hiPSC-CM line or **(B)** hiPSC-cardiomyocytes (hiPSC-CMs) harboring single nucleotide polymorphism (rs2229774) in retinoic acid receptor-γ (RARG). DMSO serves as negative control for cytotoxicity and stauro-sporine (STAURO) serves as positive control for cytotoxicity. UF DOX treatments shown in subpanels i and SPEDOX-6 treatments shown in subpanels ii. Subpanels iii show cell viability dose response curves for UF DOX or SPEDOX-6 drug treatment in either isogenic or RARG mutant hiPSC-CMs. * indicates $p<0.05$, ** indicates $p<0.01$, and *** indicates $p<0.001$ by Student's t test between DMSO and indicated condition. LD_{50} indicates drug concentration at which 50% loss in cell viability occurs.

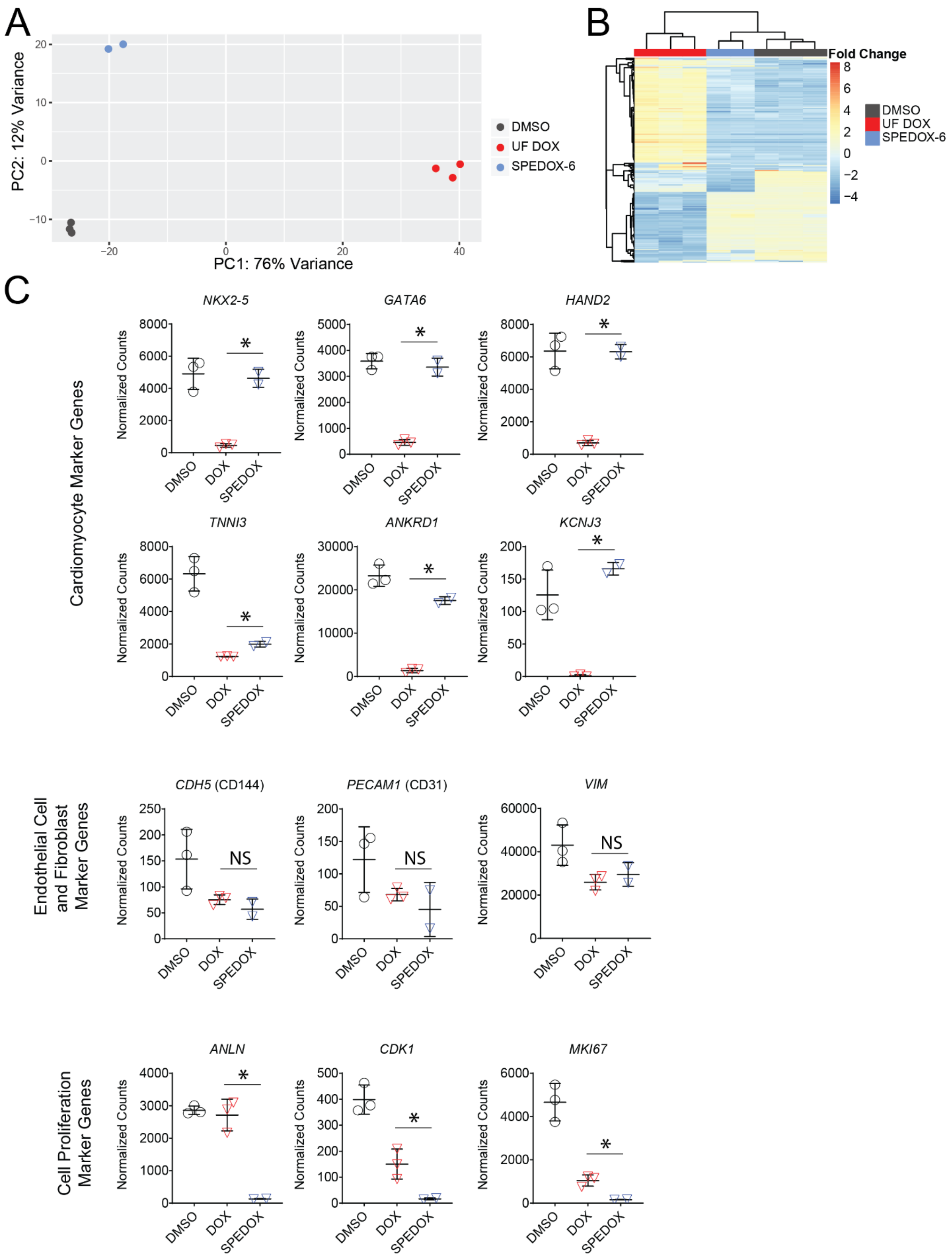
A
PC2: 12% Variance
PC1: 76% Variance
DMSO
UF DOX
SPEDOX-6
B
Fold Change
C
Cardiomyocyte Marker Genes
NKX2-5
GATA6
HAND2
TNNI3
ANKRD1
KCNJ3
Endothelial Cell and Fibroblast Marker Genes
CDH5 (CD144)
PECAM1 (CD31)
VIM
NS
Cell Proliferation Marker Genes
ANLN
CDK1
MKI67
Normalized Counts
DMSO
DOX
SPEDOX

Figure S4 (related to Figure 4): Bulk RNA-sequencing analysis after drug treatment in hiPSC-cardiac spheroids (hiPSC-CSs) indicates cell type-specific transcriptional responses in SPEDOX-6 treatment compared to unformulated doxorubicin (UF DOX). (A) Principal component analysis (PCA) plot of drug-treated cardiac spheroid RNA-seq samples. Treatment was for 48 hours. UF DOX added at 5 µM and SPEDOX-6 added at 5 µM UF DOX equivalent dose. N=3 independent experiments for DMSO and UF DOX. N=2 independent experiments for SPEDOX-6. **(B)** Heatmap of drug-treated cardiac spheroid samples indicating clustering by condition. **(C)** Representative genes indicating the cell type-specific impact of drugs on hiPSC-CMs (*NKX2-5, GATA6, HAND2, TNNI3, ANKRD1, KCNJ3*), hiPSC-ECs (*CDH5, PECAM1*), and hiPSC-CFs (*VIM*). Other genes of interest include proliferation markers *ANLN, CDK1* and *MKI67*. * indicates $p<0.05$ determined by one-way ANOVA with Tukey's post hoc test. NS indicates not significant.

SUPPLEMENTAL EXPERIMENTAL PROCEDURES

hiPSC Culture. All hiPSC cultures were maintained at 37°C in a Forma Steri-Cycle CO_2 incubator (ThermoFisher) with 5% CO_2. Cells were routinely maintained in mTeSR medium (STEMCELL Technologies) on 100 µg/mL Matrigel (Corning) and passaged every 3-4 days using 0.5 mM EDTA in PBS. hiPSCs were replenished daily with 2 mL mTeSR medium and routinely tested for mycoplasma using a MycoAlert Plus Kit (Lonza).

hiPSC-CM Differentiation. Healthy control hiPSCs derived in the Cedars-Sinai Regenerative Medicine Institute were differentiated into hiPSC-CMs following a chemically defined protocol with slight variations in the duration and concentration of CHIR99021 GSK3-beta inhibitor (Cayman Chemical) treatment, as described previously (Sharma et al., 2018). hiPSC-CMs were dissociated with TrypLE Select Enzyme 10X (ThermoFisher) when re-plated and re-seeded on Matrigel-coated plates.

hiPSC-EC Differentiation. hiPSC-ECs were produced following a chemically defined protocol with modulation of CHIR99021 GSK3-beta inhibitor as described previously (Liu et al., 2018). At day 12 of differentiation, ECs were sorted using magnetic activated cell sorting (MACS) and a CD144 antibody (Miltenyi Biotec). $CD144^+$ cells were then cultured and expanded with EGM2 media changes every 2 days.

hiPSC-CF Differentiation. hiPSC-CFs were produced following a chemically defined protocol with variation of CHIR99021 GSK3-beta inhibitor. Concentrations of CHIR99021 ranging from 8-12 µM were added to RPMI 1640 and B27 minus insulin medium for days 0-2 on hiPSCs. From days 2-3, the medium was substituted to just RPMI 1640 and B27 minus insulin (ThermoFisher), with the addition of 2 µM Wnt-C59 (ThermoFisher) at day 3 for days 3-5. EGM2 media containing 25 ng/mL FGF was then added and changed every 2 days until day 12.

hiPSC-CS Differentiation. hiPSC-CMs, hiPSC-ECs, and hiPSC-CFs were either thawed or re-plated, counted, and seeded together onto clear U-bottom 96-well plates in an 8:1:1 ratio of CMs, ECs, and CFs, respectively, for a total of 20,000 cells, as described previously (Arzt et al., 2023). A multi-lineage (hiPSC-CM, hiPSC-EC, and hiPSC-CF) medium consisting of RPMI 1640, 505 µg/mL recombinant human albumin (ScienCell Research Laboratories), 220 µg/mL L-ascorbic acid 2-phosphate (Fujifilm Wako Chemicals), 20 µg/mL insulin (ThermoFisher), 20% fetal bovine serum (ThermoFisher), and small molecules from the EGM2 media kit (Lonza) was used for seeding and changed every 2 days for 1-2 weeks for the spheroids to form and start contracting.

Immunofluorescence. Immunofluorescence was conducted according to published protocols (Sharma et al., 2017). Briefly, the hiPSCs, hiPSC-CMs, hiPSC-ECs, and hiPSC-CFs were fixed with 4% PFA and stained with selected antibodies at 1:200 dilution (Origene, Abcam). Cells were washed with phosphate buffer saline (PBS) and then incubated for 2 hours at room temperature in the dark with Alexa-Fluor-conjugated secondary antibodies (1:500, diluted in 3% BSA, ThermoFisher). Wells were washed again with PBS and incubated for 10 minutes at room temperature in the dark with DAPI (10% diluted in PBS, ThermoFisher) and imaged with an EVOS FL microscope (ThermoFisher) or BZ-X810 microscope.

Transcriptional Analysis of DMSO, UF DOX, and SPEDOX-6-treated hiPSC-CSs. Total RNA was isolated from hiPSC-CSs treated with DMSO, UF DOX (5 µM), and SPEDOX-6 (5 µM UF DOX equivalent) using a RNeasy Mini Kit (QIAGEN) with on-column DNase digestion. Nine spheroids were treated per condition for 48 hours at 40,000 cells per spheroid, following previously published ratios of hiPSC-CMs, hiPSC-ECs, and hiPSC-CFs (Arzt et al., 2023). Each spheroid was washed with 200 µL PBS and then followed by a wash with 350 µL Buffer RLT containing β-mercaptoethanol and DTT to lyse the cells. Still in Buffer RLT, 3 spheroids from 1 condition were then transferred to an Eppendorf tube for a single sample replica. 3 replicates of 3 spheroids per condition was generated, or 120,000 cells per replicate. The contents of each tube were pipetted up and down and centrifuged at 8000 x g for 3 minutes. The supernatant was then collected for further processing. 350 µL 70% ethanol was added per tube and the contents were transferred to RNeasy MinElute spin columns inside 2 mL collection tubes which were centrifuged for 15 seconds at 8000 x g. Buffer RW1 was then added to each column at 350 µL and spun for 15 seconds at 8000 x g. A mixture of Buffer RDD and DNase 1 was then added to each column at 80 µL and incubated

at room temperature for 15 minutes. An additional 350 μL Buffer RW1 was added to each collection tube and centrifuged at 8000 x g for 15 seconds. A final addition of 500 μL 80% ethanol was added to each column and centrifuged at 8000 x g for 2 minutes. After transferring the spin column to a new collection tube, each column was spun at 5 minutes at full speed. The columns were transferred to a 1.5 mL tube and 14 μL RNase free water was added to the center of each column followed by centrifugation at full speed for 1 minute to elute the RNA. After a verification of the RNA yield of each tube via a NanoDrop 2000 Spectrophotometer (ThermoFisher), the samples were submitted to the Cedars-Sinai Genomics Core for ultra-low input total RNA sequencing. RNA integrity was checked with an Agilent 2100 Bioanalyzer and only samples with an RNA integrity (RIN) score >9 were selected for cDNA library construction. Libraries were prepared with SMART-Seq v4 Ultra Low Input RNA Kit (Takara) and sequenced on a NextSeq 500 (Illumina) using single-end 75 bp reads at the Cedars-Sinai Genomics Core. Demultiplexing and conversion of raw sequencing data to FASTQ was performed with Illumina bcl2fastq software. FastQC was used to assess sequence quality and reads were aligned to the GRCh38 genome assembly (GENCODE 28) using Salmon version 0.11.3. Gene-level read counts were quantified with Salmon, and differential gene expression and normalization was subsequently performed using DESeq2. Data from this project is available at Gene Expression Omnibus (GEO) under accession number GSE235470.

SUPPLEMENTAL REFERENCES

Arzt, M., Pohlman, S., Mozneb, M., and Sharma, A. (2023). Chemically Defined Production of Tri-Lineage Human iPSC-Derived Cardiac Spheroids. Current protocols *3*, e767.

Liu, C., Cheng, L., Chen, C.L., and Sayed, N. (2018). Generation of Endothelial Cells from Human Induced Pluripotent Stem Cells. Bio-protocol *8*, e3086.

Sharma, A., Burridge, P.W., McKeithan, W.L., Serrano, R., Shukla, P., Sayed, N., Churko, J.M., Kitani, T., Wu, H., Holmstrom, A.*, et al.* (2017). High-throughput screening of tyrosine kinase inhibitor cardiotoxicity with human induced pluripotent stem cells. Science translational medicine *9*.

Sharma, A., Toepfer, C.N., Ward, T., Wasson, L., Agarwal, R., Conner, D.A., Hu, J.H., and Seidman, C.E. (2018). CRISPR/Cas9-Mediated Fluorescent Tagging of Endogenous Proteins in Human Pluripotent Stem Cells. Current protocols in human genetics *96*, 21 11 21-21 11 20.
