# Supplementary material for: Protein-encapsulated doxorubicin reduces cardiotoxicity in hiPSC-cardiomyocytes and cardiac spheroids while maintaining anticancer efficacy
Source: Stem Cell Reports. 2023 Aug 31;18(10):1913–24. doi: 10.1016/j.stemcr.2023.08.005 (PMC10656302; doi:10.1016/j.stemcr.2023.08.005)
Supplement: Document S2. Article plus supplemental information [file mmc6.pdf]

Stem Cell Reports
Report

OPEN ACCESS

Protein-encapsulated doxorubicin reduces cardiotoxicity in hiPSC-cardiomyocytes and cardiac spheroids while maintaining anticancer efficacy

Madelyn Arzt,[1,2,3,4,13] Bowen Gao,[5,13] Maedeh Mozneb,[1,2,3,4] Stephany Pohlman,[1,2,3,4,6] Romina B. Cejas,[7,8] Qizhi Liu,[5] Faqing Huang,[9] Changjun Yu,[10,11] Yi Zhang,[3] Xuemo Fan,[12] Amelia Jenkins,[1,2,3,4] Armando E. Giuliano,[5] Paul W. Burridge,[7,8] Xiaojiang Cui,[4,5,*] and Arun Sharma[1,2,3,4,14,*]
[1]Board of Governors Regenerative Medicine Institute, Cedars-Sinai Medical Center, Los Angeles, CA, USA
[2]Smidt Heart Institute, Cedars-Sinai Medical Center, Los Angeles, CA, USA
[3]Department of Biomedical Sciences, Cedars-Sinai Medical Center, Los Angeles, CA, USA
[4]Samuel Oschin Comprehensive Cancer Institute, Cedars-Sinai Medical Center, Los Angeles, CA, USA
[5]Department of Surgery, Samuel Oschin Comprehensive Cancer Institute, Cedars-Sinai Medical Center, Los Angeles, CA, USA
[6]CIRM Bridges to Stem Cell Research Program, California State University, Channel Islands, CA, USA
[7]Department of Pharmacology, Northwestern University Feinberg School of Medicine, Chicago, IL, USA
[8]Center for Pharmacogenomics, Northwestern University Feinberg School of Medicine, Chicago, IL, USA
[9]Department of Chemistry and Biochemistry, University of Southern Mississippi, Hattiesburg, MS, USA
[10]Division of Chemistry & Chemical Engineering, California Institute of Technology, Pasadena, CA, USA
[11]Sunstate Biosciences LLC, Monrovia, CA, USA
[12]Department of Pathology, Cedars-Sinai Medical Center, Los Angeles, CA, USA
[13]These authors contributed equally
[14]Twitter: @ArunSharmaPhD
*Correspondence: xiaojiang.cui@cshs.org (X.C.), arun.sharma@cshs.org (A.S.)
https://doi.org/10.1016/j.stemcr.2023.08.005

SUMMARY

The chemotherapeutic doxorubicin (DOX) detrimentally impacts the heart during cancer treatment. This necessitates development of non-cardiotoxic delivery systems that retain DOX anticancer efficacy. We used human induced pluripotent stem cell-derived cardiomyocytes (hiPSC-CMs), endothelial cells (hiPSC-ECs), cardiac fibroblasts (hiPSC-CFs), multi-lineage cardiac spheroids (hiPSC-CSs), patient-specific hiPSCs, and multiple human cancer cell lines to compare the anticancer efficacy and reduced cardiotoxicity of single protein encapsulated DOX (SPEDOX-6), to standard unformulated (UF) DOX. Cell viability assays and immunostaining in human cancer cells, hiPSC-ECs, and hiPSC-CFs revealed robust uptake of SPEDOX-6 and efficacy in killing these proliferative cell types. In contrast, hiPSC-CMs and hiPSC-CSs exhibited substantially lower cytotoxicity during SPEDOX-6 treatment compared with UF DOX. SPEDOX-6-treated hiPSC-CMs and hiPSC-CSs maintained their functionality, as indicated by sarcomere contractility assessment, calcium imaging, multi-electrode arrays, and RNA sequencing. This study demonstrates the potential of SPEDOX-6 to alleviate cardiotoxic side effects associated with UF DOX, while maintaining its anticancer potency.

INTRODUCTION

Small molecule anthracyclines, such as doxorubicin (DOX), are used to combat multiple cancer types (Moslehi, 2016; Rhee et al., 2020; Sayed et al., 2019). However, DOX infiltrates the heart during treatment, generating reactive oxygen species, causing DNA and mitochondrial damage, and inhibiting topoisomerase, leading to transcriptional alterations and apoptosis (Burridge et al., 2016; Sayed et al., 2019). DOX can also trigger calcium overload, leading to arrhythmias (Burridge et al., 2016). After DOX treatment, patients face cardiovascular complications with acute and chronic presentations. Acute DOX toxicity, consisting of cardiac rhythm disturbances or hypotension, typically resolves once treatment is completed (Sayed et al., 2019). However, chronic toxicity leading to heart failure can be observed years after treatment (Sayed et al., 2019). There remains an interest in predicting which patients will be affected by cardiotoxicity before beginning treatment with DOX or the degree to which DOX could cause cardiac injury (Burridge et al., 2016).

Such severe side effects present the need to reduce the DOX cardiotoxicity by limiting its access to cardiac cells, while maintaining its anti-tumor efficacy. Incorporating DOX into bioengineered systems, uncoupling DOX-induced DNA damage from chromatin damage, and using nanoparticles and biopolymers have improved the pharmacokinetics and tumor-specific delivery of DOX (Kratz, 2008; Qiao et al., 2020). However, there have been challenges and significant variability during treatment with such approaches. Human serum albumin (HSA) binds many natural ligands and serves as a major nutrient source that is rapidly consumed and metabolized by proliferative cancer cells. These properties make HSA an ideal carrier protein to selectively target DOX to cancer cells and reduce the off-target cardiotoxicity of DOX (Yu et al., 2020).

Many drug cardiotoxicity testing platforms use animal models, which exhibit significant differences in cardiac physiology when compared with the human heart (Sharma et al., 2017). While ideal for assessing drug cardiotoxicity, the use of primary human cardiomyocytes remains limited, based on the difficulty of obtaining cells for large-scale

This is an open access article under the CC BY license (http://creativecommons.org/licenses/by/4.0/).

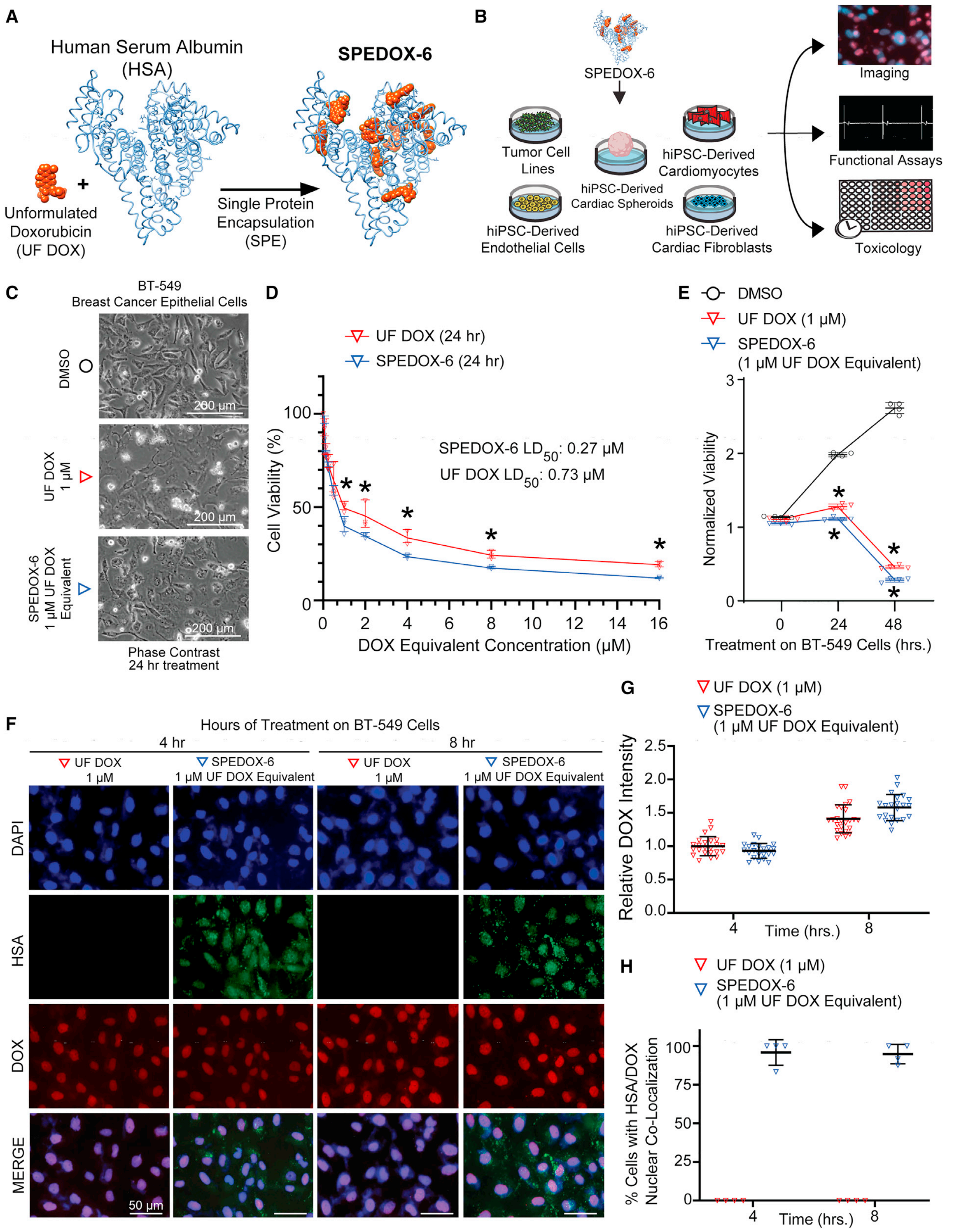

(legend on next page)

cardiotoxicity screening (Sayed et al., 2019; Sharma et al., 2017). Human induced pluripotent stem cells (hiPSCs) have emerged as an alternative platform for mass-producing human cardiovascular cells via targeted cellular differentiation. Also, hiPSC-derived cardiomyocytes (hiPSC-CMs), endothelial cells (hiPSC-ECs), and cardiac fibroblasts (hiPSC-CFs) are patient specific and can recapitulate human physiology and cardiovascular disease phenotypes *in vitro* (Sharma et al., 2017). Additionally, advances in tissue engineering and organoid biology have enabled production of multi-lineage, hiPSC-derived cardiac microtissues composed of CMs, ECs, and CFs, enabling more realistic, three-dimensional (3D) modeling of cardiovascular disease (Giacomelli et al., 2017, 2020). Finally, previous studies have shown that hiPSCs can elucidate patient-specific phenotypes associated with DOX-induced cardiotoxicity (Burridge et al., 2016). Thus, targeted drug development, preclinical hiPSC modeling, and new 3D model systems may enable safer anticancer treatments (Asnani et al., 2021).

We developed a novel DOX delivery system, single protein encapsulated DOX (SPEDOX-6), in which multiple DOX molecules are encompassed by a single HSA protein without covalent conjugation. Using a mouse model, we have previously shown that SPEDOX-6 holds favorable anti-tumor activity and pharmacokinetic profiles superior to unformulated DOX (UF DOX) (Yu et al., 2020). Even with a 4-fold increase in the maximum tolerated UF DOX equivalent dose, SPEDOX-6 did not show increased systemic toxicity, reflected in insignificant weight loss in the treated mice when compared with UF DOX. Because of its encapsulation with HSA, SPEDOX-6 may reduce non-specific DOX uptake by non-cancerous tissues, but its cardiac cellular impacts have not been examined. Thus, we seek to evaluate the cardiotoxic side effects of SPEDOX-6 *in vitro*. We hypothesized that SPEDOX-6 can selectively target proliferative human cancer cells *in vitro*, while reducing off-target toxicity on non-proliferative hiPSC-CMs. Through cellular cytotoxicity and functional assessments, we compared the cardiotoxicity and anti-tumor efficacy of UF DOX and SPEDOX-6 in hiPSC-CMs, hiPSC-ECs, hiPSC-CFs, multi-lineage hiPSC-derived cardiac spheroids (hiPSC-CSs), patient-specific hiPSCs, and human cancer cell lines.

RESULTS

Encapsulation of DOX into HSA and anticancer efficacy against human cancer cells

UF DOX was encapsulated into HSA as per previous studies, at a ratio of 9 UF DOX:1 HSA, to obtain purified SPEDOX-6 (Figure 1A) (Yu et al., 2020). We subjected *in vitro* model systems to SPEDOX-6, including hiPSC-CMs, hiPSC-ECs, hiPSC-CFs, multi-lineage hiPSC-CSs, and multiple human cancer cell lines (Figure 1B). We then conducted imaging, functional, and toxicology assays to evaluate anticancer and cardiotoxic impacts of SPEDOX-6.

To confirm the anti-tumor effects of SPEDOX-6, we first subjected human cancer cells to both UF DOX and SPEDOX-6. In BT-549 breast cancer cells, SPEDOX-6 elicited similar detrimental impacts to UF DOX on cell viability within hours of treatment (Figures 1C–1E). Using immunostaining, we confirmed that SPEDOX-6, in a similar manner as HSA, was able to internalize within human breast cancer cells within hours of treatment (Figures 1F–1H and S1A). Immunofluorescence staining also indicated nuclear accumulation of SPEDOX-6 within hours of treatment on cancer cells, at a comparable level to UF DOX. HSA alone did not exhibit cytotoxicity (Figure S1B) and SPEDOX-6 also exhibited comparable anticancer efficacy to UF DOX in HT-1080 fibrosarcoma cells (Figures S1C and S1D). Taken together, these results suggest that the anticancer effect of SPEDOX-6 is comparable with UF DOX and, like exogenous HSA carrier protein, SPEDOX-6 can rapidly internalize in proliferative cancer cells.

Figure 1. SPEDOX-6 exhibits comparable anticancer efficacy to UF DOX on human breast cancer epithelial cells *in vitro*
(A) Schematic illustrating SPE methodology. A single HSA molecule encapsulates multiple DOX molecules to form SPEDOX-6.
(B) Experimental workflow of *in vitro* systems being subjected to SPEDOX-6.
(C) Phase contrast imaging of BT-549 breast cancer epithelial cells subjected to DMSO, UF DOX, or SPEDOX-6 for 24 h.
(D) CellTiter-Glo assay for assessing cellular viability in BT-549 cells after 24 h of SPEDOX-6 or UF DOX treatment at increasing drug doses. LD_{50}, drug concentration at which 50% of cells have died. $^*p < 0.05$ by Student t test. n = 3 independent experiments.
(E) CellTiter-Glo assay for assessing cellular viability in BT-549 cells after 24 and 48 h of DMSO, UF DOX, or SPEDOX-6 treatment. Higher values indicate higher cell viability. $^*p < 0.05$ between DMSO and other groups, determined by one-way ANOVA with Tukey's post hoc test. n = 4 independent experiments.
(F) Immunofluorescence of BT-549 cells after 4 and 8 h of UF DOX or SPEDOX-6 treatment. HSA represents human serum albumin and DOX represents doxorubicin accumulation in BT-549 cells.
(G) Quantification of nuclear DOX signal intensity corresponding with immunofluorescence in (F). n = 22 technical replicates (images). Error bars represent SD.
(H) Quantification of percentage of cells with nuclear co-localization of HSA and DOX, corresponding with immunofluorescence in (F). n = 4 technical replicates (images). Error bars represent SD.

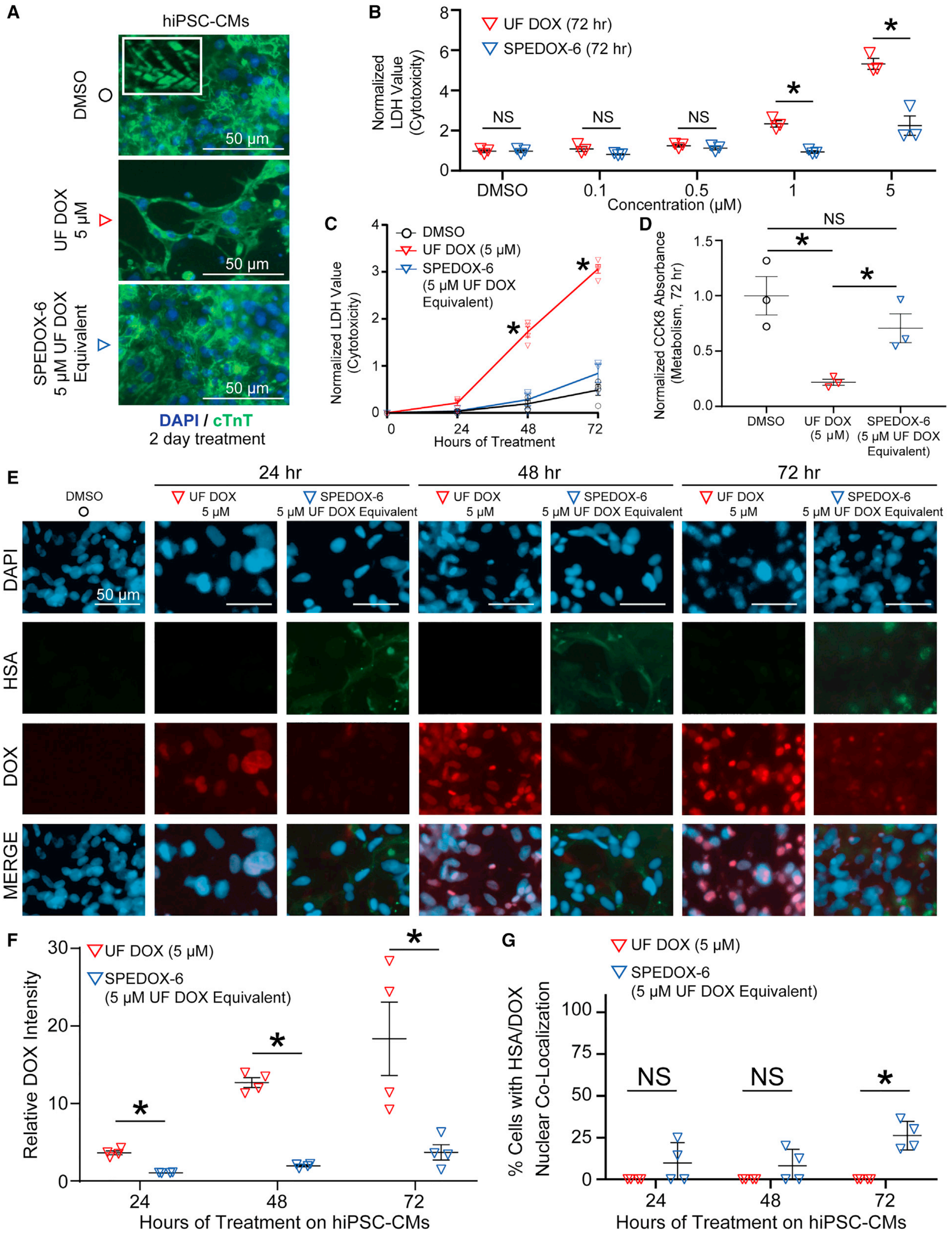

(legend on next page)

SPEDOX-6 exhibits reduced cytotoxicity on hiPSC-CMs

To evaluate the cardiotoxicity associated with SPEDOX-6, we subjected hiPSC-CMs to UF DOX or SPEDOX-6. The hiPSCs from multiple cell lines expressed pluripotency markers before differentiation into hiPSC-CMs (Figure S2A), and high-performance liquid chromatography results suggested that SPEDOX-6 is stable in CM medium (Figure S2B). The hiPSC-CMs were then subjected to SPEDOX-6 treatment at multiple drug concentrations and timepoints (Figure 2). Visualization of UF DOX and SPEDOX-6-treated hiPSC-CMs demonstrated loss of cell integrity after UF DOX treatment, but not after SPEDOX-6 treatment (Figure 2A). Lactate dehydrogenase (LDH) cytotoxicity assays confirmed that SPEDOX-6 induced significantly lower cytotoxicity than UF DOX in hiPSC-CMs during a dose response (Figure 2B) and a multi-day time course (Figure 2C). The CCK8 cell metabolism and viability assay also demonstrated that SPEDOX-6-treated hiPSC-CMs maintained their viability and metabolism at a higher level than UF DOX-treated hiPSC-CMs (Figure 2D). Immunofluorescence staining on hiPSC-CMs was able to confirm nuclear accumulation of UF DOX, but showed significantly less nuclear accumulation of SPEDOX-6 and HSA (Figures 2E–2G). HSA treatment alone on hiPSC-CMs did not enhance cytotoxicity to levels above DMSO (Figure S2C). Next, since multiple studies have demonstrated a strong genetic component to anthracycline cardiotoxicity (Kim et al., 2022; Knowles et al., 2018; Magdy et al., 2022), we also conducted cytotoxicity analyses on hiPSC-CMs derived from a patient-specific hiPSC line harboring a single nucleotide polymorphism (rs2229774) in retinoic acid receptor-γ (RARG) (Magdy et al., 2021), as well as a corresponding isogenic control hiPSC-CM line. The patient-specific RARG mutant hiPSC-CMs, which show enhanced susceptibility to DOX-induced cardiotoxicity, exhibited less cytotoxicity after SPEDOX-6 treatment in comparison with UF DOX treatment (Figure S3). Taken together, these results suggest that SPEDOX-6 elicits markedly less cytotoxicity than UF DOX in hiPSC-CMs.

hiPSC-CMs maintain contractile functionality after SPEDOX-6 treatment

We next evaluated the functional outcomes of SPEDOX-6 treatment on hiPSC-CM contractility. SPEDOX-6 treatment on hiPSC-CMs maintained hiPSC-CM beat rate at a significantly higher level than UF DOX over multiple days of drug treatment (Figure 3A, Video S1). Maintenance of hiPSC-CM contractile function after SPEDOX-6 treatment was also confirmed using multielectrode arrays (Figures 3B and 3C) and automated, edge detection-based analysis of cardiomyocyte contractility videos using MUSCLEMOTION software (Sala et al., 2018) (Figure S2D). Fluorescent reporter hiPSC-CMs harboring GFP at the sarcomere Z line (ACTN2-GFP) were also subjected to SPEDOX-6 and UF DOX treatment (Figure 3D). ACTN2-GFP hiPSCs exhibited sarcomere disassembly more rapidly after UF DOX treatment than after SPEDOX-6 treatment. After treatment with UF DOX or SPEDOX-6, the contractility and displacement of single sarcomeres in ACTN2-GFP hiPSC-CMs was evaluated using SarcTrack sarcomere tracking software (Toepfer et al., 2019). Analyses indicated that sarcomere displacement, contraction, and relaxation were restored closer to DMSO levels after SPEDOX-6 treatment, than after UF DOX treatment (Figures 3E and 3F, Video S2). A third hiPSC-CM line, harboring an endogenous fluorescent calcium reporter (Huebsch et al., 2015), was used for live calcium imaging of UF DOX and SPEDOX-6-treated hiPSC-CMs. These fluorescent calcium reporter (WTC-GCaMP) hiPSC-CMs exhibited improved

Figure 2. SPEDOX-6 induces less cytotoxicity than UF DOX in hiPSC-CMs

(A) Immunofluorescence of hiPSC-CMs subjected to DMSO, UF DOX, or SPEDOX-6 for 2 days. Cardiac troponin T (cTnT) represents a CM-specific protein marking the striated cardiac sarcomere (inset).

(B) LDH dose response assay for assessing cellular cytotoxicity in hiPSC-CMs after 3 days of DMSO, UF DOX, or SPEDOX-6 treatments at increasing drug doses. Higher values indicate higher drug-induced cytotoxicity. *$p < 0.05$ by Student *t* test. NS, non-significance. n = 3 independent experiments.

(C) LDH time course assay for assessing cellular cytotoxicity in hiPSC-CMs cells after 3 days of DMSO, UF DOX, or SPEDOX-6 treatments. Higher values indicate higher drug-induced cytotoxicity. *$p < 0.05$ between DMSO and other groups, determined by one-way ANOVA with Tukey's post hoc test. n = 4 independent experiments.

(D) CCK8 assay for assessing cellular metabolic output in hiPSC-CMs treated with DMSO, UF DOX, or SPEDOX-6 for 72 h. Higher values indicate higher cellular metabolic output. NS indicates non-significance. *$p < 0.05$ determined by one-way ANOVA with Tukey's post hoc test. n = 3 independent experiments.

(E) Immunofluorescence of SPEDOX-6 treatment on hiPSC-CMs for up to 3 days. HSA represents human serum albumin and DOX represents doxorubicin accumulation in hiPSC-CMs.

(F) Quantification of nuclear DOX signal intensity after UF DOX or SPEDOX-6 treatment. *$p < 0.05$ by Student *t* test. n = 4 independent experiments.

(G) Quantification of percentage of cells with nuclear co-localization of HSA and DOX. *$p < 0.05$ by Student *t* test. n = 4 independent experiments.

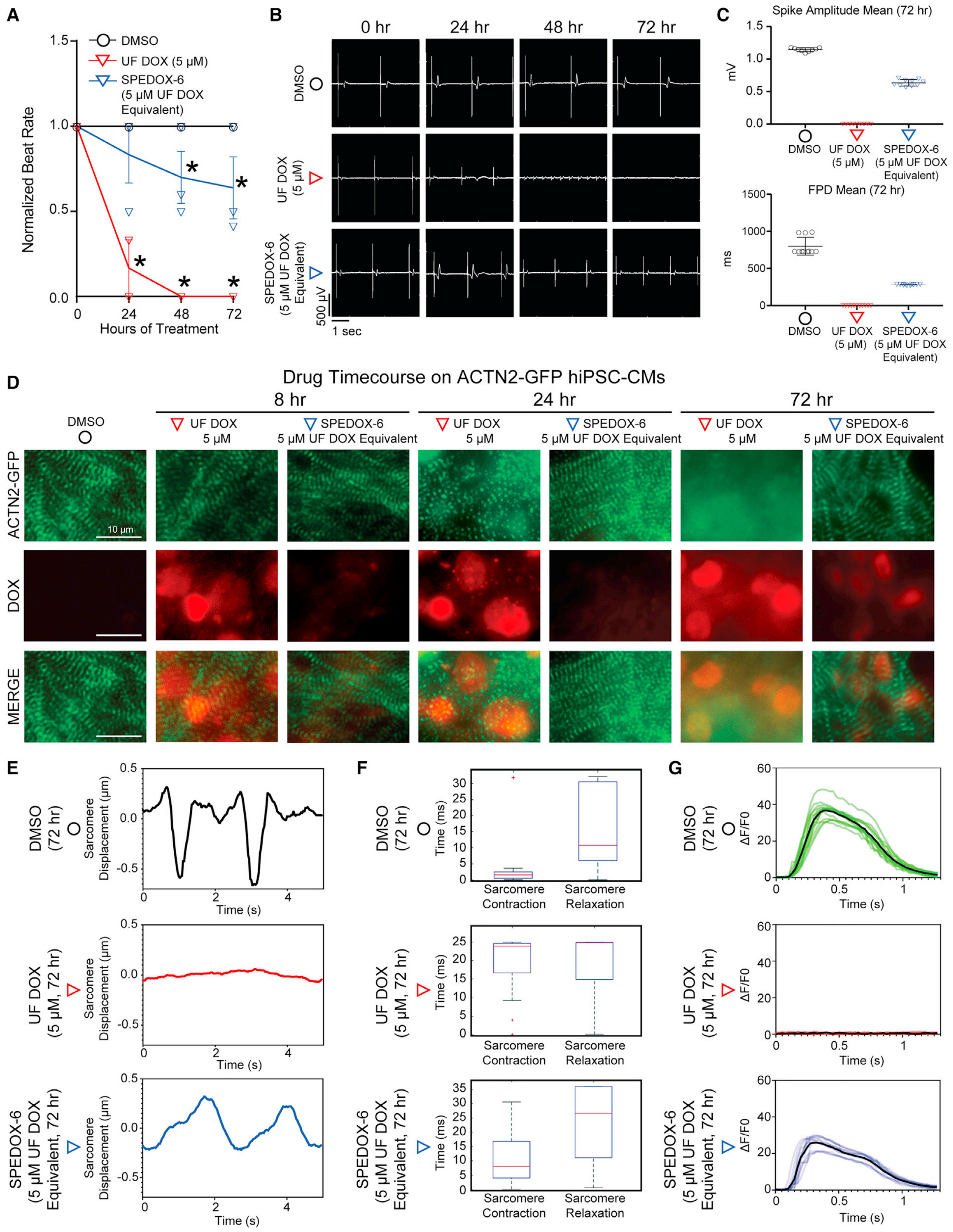

(legend on next page)

calcium handling in response to SPEDOX-6 treatment compared with UF DOX treatment (Figure 3G, Video S2). Taken together, these results suggest that SPEDOX-6 elicits less functional cardiotoxicity than UF DOX in hiPSC-CMs.

Cardiotoxicity of SPEDOX-6 on hiPSC-ECs and hiPSC-CFs

To determine the cardiotoxic impact of SPEDOX-6 on other cardiovascular cell types, we subjected hiPSC-ECs and hiPSC-CFs to SPEDOX-6 and UF DOX (Figure 4). hiPSC-CMs typically show limited proliferation after day 30 of differentiation, unless subjected to exogenous stimulation (Sharma et al., 2017). In contrast, hiPSC-ECs and hiPSC-CFs are proliferative and can be expanded *in vitro*. hiPSC-ECs expressing canonical endothelial protein markers (Figure 4A) did not show a significant difference in cytotoxicity between UF DOX or SPEDOX-6 treatment (Figure 4B). Unlike hiPSC-CMs, immunofluorescence staining demonstrated that hiPSC-ECs were able to internalize SPEDOX-6 and UF DOX equally well (Figures 4C–4E). Similarly, hiPSC-CFs expressing canonical fibroblast protein markers (Figure 4F) exhibited comparable cytotoxicity in response to UF DOX or SPEDOX-6 drug treatment (Figure 4G). Immunostaining indicated comparable uptake and internalization of SPEDOX-6 and UF DOX in hiPSC-CFs (Figures 4H–4J). Taken together, these results suggest that proliferative hiPSC-ECs and hiPSC-CFs exhibit similar toxic effects from SPEDOX-6 as UF DOX.

SPEDOX-6 exhibits reduced cytotoxicity on multi-lineage hiPSC-derived cardiac spheroids harboring hiPSC-CMs, -ECs, and -CFs

To determine the influence of cellular contact and co-culture on SPEDOX-6-induced cardiotoxicity, we used hiPSC-CSs composed of hiPSC-CMs, hiPSC-ECs, and hiPSC-CFs co-cultured in a 3D, spherical, self-aggregating format (Arzt et al., 2023). hiPSC-CSs were composed of 20,000 cells total in an 8:1:1 ratio of CMs, ECs, and CFs respectively, comparable with previous studies (Giacomelli et al., 2020), and exhibited rhythmic contractility (Video S3). We established multiple versions of the hiPSC-CS model, including hiPSC-CSs harboring fluorescent reporter CMs and ECs (Figure 4K, Video S4). The hiPSC-CSs subjected to SPEDOX-6 exhibited lower cytotoxicity in comparison with hiPSC-CSs subjected to UF DOX (Figure 4L), similar to what was observed with two-dimensional (2D) hiPSC-CMs. SPEDOX-6 treatment on hiPSC-CSs also maintained contractility at a higher level than treatment with UF DOX, as measured by edge detection methodologies (Figures 4M and 4N). However, both contractile force and beat rate of the hiPSC-CSs were still altered by SPEDOX-6 (Figure 4M). Fluorescent calcium reporter hiPSC-CMs from the aforementioned WTC-GCaMP hiPSC line were also used to make hiPSC-CSs (Video S4). These fluorescent calcium reporter hiPSC-CSs exhibited improved calcium handling in response to SPEDOX-6 treatment in comparison with UF DOX treatment (Figure S2E).

Bulk RNA sequencing was also performed on CSs treated with DMSO, UF DOX, or SPEDOX-6 (Figure S4), initially showing that transcriptomic samples clustered by treatment condition (Figures S4A and S4B). Additionally, the expression of cardiac markers such as *NKX2-5*, *HAND2*, *TNNI3*, *GATA6*, *KCNJ3*, and *ANKRD1* were significantly higher in SPEDOX-6-treated CSs in comparison with UF DOX-treated CSs, further indicating reduced CM-specific toxicity in SPEDOX-6-treated CSs (Figure S4C). In contrast, the expression of endothelial markers *CDH5* and *PECAM1*, as well as fibroblast marker *VIM*, were not significantly different between UF DOX and SPEDOX-6 conditions, indicating no difference in EC- or CF-specific toxicity

Figure 3. SPEDOX-6 induces less functional toxicity than UF DOX in hiPSC-CMs

(A) hiPSC-CM beat rate normalized to DMSO after 3 days of UF DOX or SPEDOX-6 treatment. See Video S1. *$p < 0.05$ between DMSO and other groups, determined by one-way ANOVA with Tukey's post hoc test. n = 4 independent experiments.

(B) Representative field potential recordings from contracting hiPSC-CMs in multielectrode arrays treated with DMSO, UF DOX, or SPEDOX-6 for up to 72 h.

(C) Average spike amplitude mean and field potential duration (FPD) mean from field potential recordings of contracting hiPSC-CMs in multielectrode arrays treated with DMSO, UF DOX, or SPEDOX-6 for up to 72 h, corresponding with (B). n = 9 technical replicates. Error bars represent SD.

(D) Live fluorescence imaging of ACTN2-GFP hiPSC-CMs subjected to DMSO, UF DOX, or SPEDOX-6 for up to 72 h. α-actinin (green) represents a cardiomyocyte-specific protein marking the striated cardiac sarcomeres in live hiPSC-CMs. DOX (red) shows DOX intracellular accumulation.

(E) SarcTrack dataset showing representative sarcomere displacement timegraphs of ACTN2-GFP hiPSC-CMs treated with DMSO, UF DOX, or SPEDOX-6 for 72 h. See Video S2.

(F) SarcTrack-based quantification of sarcomere displacement during the hiPSC-CM contraction cycle in ACTN2-GFP hiPSC-CMs treated with DMSO, UF DOX, or SPEDOX-6 for 72 h. See Video S2. n = 10, 29, and 22 sarcomeres were detected for DMSO, UF DOX, and SPEDOX-6 conditions, respectively.

(G) Calcium imaging timegraphs of WTC-GCaMP hiPSC-CMs treated with DMSO, UF DOX, or SPEDOX-6 for 72 h. $\Delta F/F0$ compares the change of the fluorescence intensity to the baseline fluorescence intensity before the contraction. See Video S2.

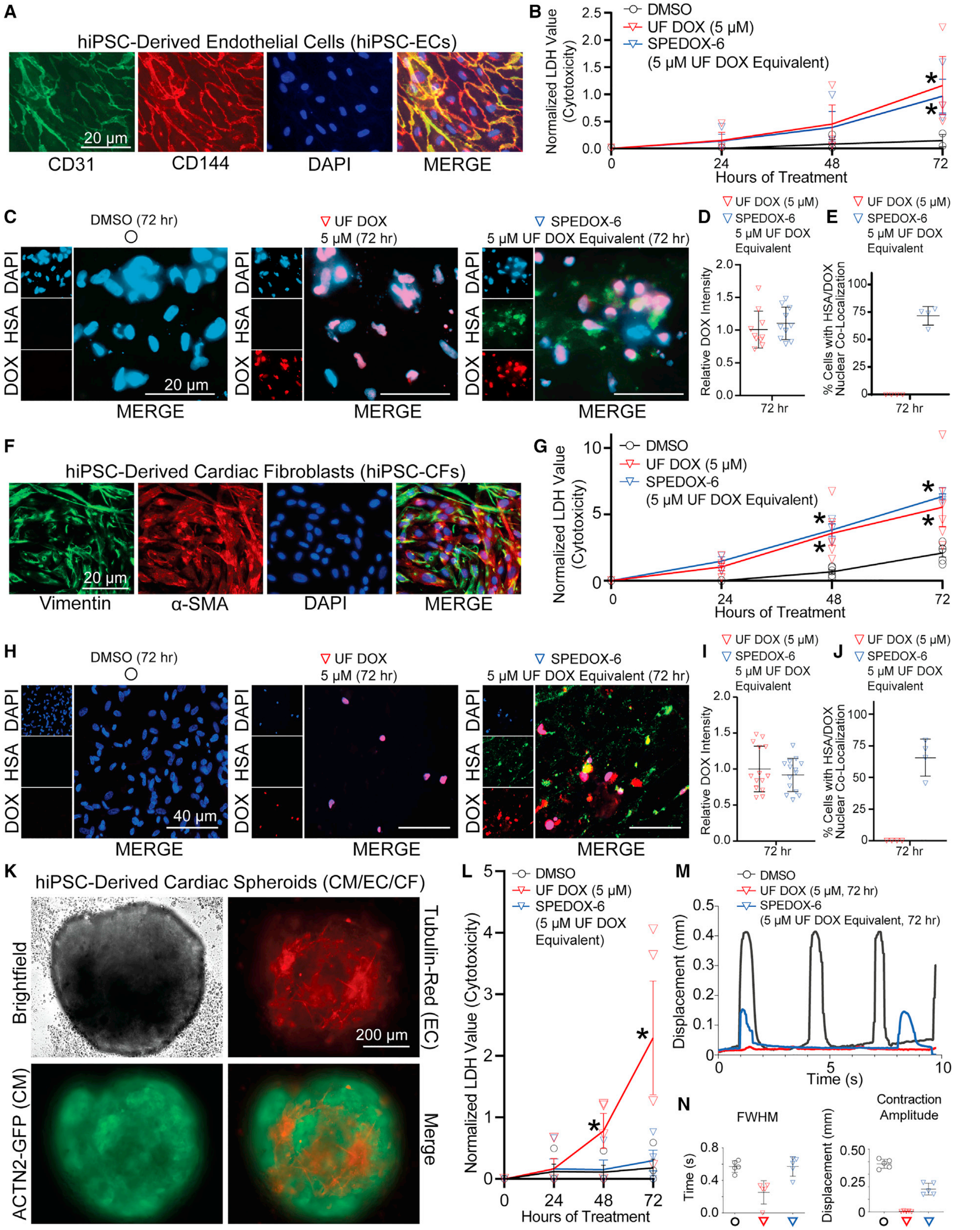

(legend on next page)

between SPEDOX-6- and UF DOX-treated CSs (Figure S4C). The expression of cell proliferation markers *ANLN*, *CDK1*, and *MKI67* were significantly lower in SPEDOX-6-treated CSs in comparison with UF DOX-treated CSs (Figure S4C).

Taken together, these hiPSC-CS results show that SPEDOX-6 induces less cytotoxicity and functional cardiotoxicity than UF DOX in a 3D, multi-lineage, cardiovascular co-culture system. RNA sequencing data from drug-treated CSs further suggest that SPEDOX-6 induces cell-type-specific cytotoxicity, selectively targeting proliferative cells as opposed to non-proliferative cardiomyocytes.

DISCUSSION

We demonstrate that SPEDOX-6 retains the anticancer efficacy of UF DOX, while minimizing its cardiotoxicity. Human breast cancer and fibrosarcoma cell lines confirm the ability of SPEDOX-6 to accumulate and elicit detrimental impacts in proliferative tumor cell models. Additionally, the decrease in cytotoxicity and enhanced contractility induced by SPEDOX-6 in comparison with UF DOX in hiPSC-CMs and hiPSC-CSs reflects a reduced cardiomyocyte-specific uptake and toxicity with SPEDOX-6. Notably, treatment with HSA alone did not induce cytotoxicity, suggesting that HSA may act as a buffer in this system. HSA uptake was notably higher in the tested cancer cell lines compared with hiPSC-CMs based on the selective design of SPEDOX-6 to target and accumulate in proliferative cells, which rapidly uptake HSA protein as an energy source. This is also reflected in the enhanced cytotoxicity and internalization of SPEDOX-6 in endothelial cells and cardiac fibroblasts, which express higher levels of known albumin binding proteins (e.g., SPARC, FcRn) than cardiomyocytes, based on recent hiPSC RNA sequencing datasets (Giacomelli et al., 2020) and other single cell RNA sequencing datasets (Litvinukova et al., 2020; Merlot et al., 2014). Our own RNA sequencing dataset from UF DOX- and SPEDOX-6-treated cardiac spheroids also reflected the selective ability of SPEDOX-6 to target proliferative cell types instead of non-proliferative cardiomyocytes. We also observed a significantly decreased expression of the proliferation markers *ANLN*, *CDK1*, and *MKI67* in SPEDOX-6-treated cardiac spheroids when compared with UF DOX-treated cardiac spheroids. Therefore, we believe that a major advantage of using SPEDOX-6 over UF DOX is the mechanism of its selective uptake, leading to decreased off-target cardiomyocyte toxicity and enhanced on-target anticancer efficacy. We also evaluated hiPSC-CMs harboring a patient-specific, genetic predilection for DOX-induced cardiac injury and showed that these DOX-sensitive cells exhibit lower cardiotoxicity after SPEDOX-6 treatment compared with UF DOX treatment. Altogether, we demonstrate the potential of an HSA-targeted,

Figure 4. SPEDOX-6 induces comparable cytotoxicity to UF DOX in hiPSC-ECs and hiPSC-CFs, but reduced cardiotoxicity in multi-lineage hiPSC-derived cardiac spheroids (hiPSC-CSs)

(A) Immunofluorescence staining of hiPSC-ECs illustrates vascular endothelial markers CD31 (PECAM1) and CD144 (VE-Cadherin).

(B) LDH cytotoxicity assay demonstrates no significant difference in toxicity in hiPSC-ECs treated for 3 days with either UF DOX or SPEDOX-6. *$p < 0.05$ between DMSO and other groups, determined by one-way ANOVA with Tukey's post hoc test. n = 3 independent experiments.

(C) Immunofluorescence demonstrates nuclear accumulation of SPEDOX-6 and DOX, as well as HSA accumulation in SPEDOX-6-treated hiPSC-EC nuclei.

(D) Quantification of nuclear DOX signal intensity corresponding to immunofluorescence in C. n = 10 technical replicates (images). Error bars represent SD.

(E) Quantification of percentage of cells with nuclear co-localization of HSA and DOX, corresponding to immunofluorescence in C. n = 4 technical replicates (images). Error bars represent SD.

(F) Immunofluorescence staining of hiPSC-CFs illustrates fibroblast markers vimentin and α-smooth muscle actin.

(G) LDH cytotoxicity assay demonstrates no significant difference in toxicity in hiPSC-CFs treated for 3 days with either UF DOX or SPEDOX-6. *$p < 0.05$ between DMSO and other groups, determined by one-way ANOVA with Tukey's post hoc test. n = 4 independent experiments.

(H) Immunofluorescence demonstrates nuclear accumulation of SPEDOX-6 and DOX, as well as HSA accumulation in SPEDOX-6-treated hiPSC-CF nuclei.

(I) Quantification of nuclear DOX signal intensity corresponding to immunofluorescence in H. n = 15 technical replicates (images). Error bars represent SD.

(J) Quantification of percentage of cells with nuclear co-localization of HSA and DOX, corresponding with immunofluorescence in H. n = 4 technical replicates (images). Error bars represent SD.

(K) Live fluorescence imaging of contractile, multi-lineage hiPSC-CSs composed of hiPSC-CMs, hiPSC-ECs, and hiPSC-CFs in an 8:1:1 ratio. CMs are labeled with ACTN2-GFP and ECs are labeled with Tubulin-RFP. See Videos S3 and S4.

(L) LDH cytotoxicity assay in hiPSC-CSs demonstrates reduced cytotoxicity after 72-h SPEDOX-6 treatment compared with UF DOX. *$p < 0.05$ between DMSO and other groups, determined by one-way ANOVA with Tukey's post hoc test. n = 4 independent experiments.

(M) Representative contractility edge displacement timegraphs of hiPSC-CSs after 72-h drug treatment.

(N) Full width at half maximum (FWHM) and amplitude of edge displacement measures of contracting hiPSC-CSs, corresponding with M. n = 5 technical replicate hiPSC-CSs. Error bars represent SD.

DOX-based treatment mechanism with potential to decrease off-target cardiotoxicity.

The results presented here validate the utility of hiPSC-derived cardiovascular cell types, 3D multi-lineage model systems, and live fluorescent reporter cell lines in drug cardiotoxicity assessment studies. The scalability of hiPSCs enables the mass production of human cardiomyocytes, endothelial cells, and cardiac fibroblasts for high-throughput drug screening assays, alleviating the dependency on animal models. However, the large-scale generation of hiPSCs and 3D cardiac spheroids can be costly and time intensive, delaying personalized drug toxicity studies for individual cancer patients. The hiPSC-CMs are also structurally, functionally, and genetically immature in comparison with adult human cardiomyocytes, especially when evaluating arrhythmias, electrophysiology, and calcium signaling in 2D (Sharma et al., 2017). Nonetheless, the widespread use of DOX and other cardiotoxic chemotherapeutic agents demonstrates the urgency to identify effective anticancer treatments that lessen damage to the heart. Given these results, SPEDOX-6 will be further investigated in clinical trials as a less cardiotoxic version of DOX that retains its anticancer efficacy. The U.S. Food and Drug Administration has green-lighted a human phase IB/IIA clinical trial for SPEDOX-6 (Investigational New Drug number 152154) and provided SPEDOX-6 an Orphan Drug Designation for treating soft tissue sarcomas. Testing should also continue to assess the broad applicability of single protein encapsulation technology to alleviate the cardiotoxicities associated with other chemotherapeutic agents, such as tyrosine kinase inhibitors. Future studies will use hiPSCs, 3D multilineage models, and human genetics to predict patient-specific susceptibilities to drug cardiotoxicity. The ability of hiPSCs to be patient-specific highlights a potential future of precision medicine for both cardiology and oncology.

EXPERIMENTAL PROCEDURES

Resource availability

Corresponding authors

Further information and requests for resources and reagents should be directed to and will be fulfilled by the corresponding authors, Arun Sharma (arun.sharma@cshs.org) and Xiaojiang Cui (Xiaojiang.cui@cshs.org).

Materials availability

This study did not generate new unique reagents.

Data and code availability

Data from this project is available at Gene Expression Omnibus (GEO: GSE235470).

Preparation of SPEDOX-6

SPEDOX-6 was prepared according to published methods (Yu et al., 2020) (Figure 1A). Good laboratory practice (GLP)-grade SPEDOX-6, manufactured by Societal CDMO San Diego, LLC was used for this study and also used for the GLP-toxicology study on SPEDOX-6, as part of investigational new drug (IND) applications to the FDA (IND #: 152154).

Drug treatment on cancer cells and cell viability/proliferation assay

BT-549 breast cancer epithelial cells, MDA-MB-231 breast cancer epithelial cells, or HT-1080 fibrosarcoma cells were seeded in 96-well plates in triplicate. Cells were treated with DMSO control, UF DOX, and SPEDOX-6 as indicated. Cell proliferation and viability assays were performed after 24- and 48-h treatments using the CellTiter-Glo luminescence assay kit (Promega) according to manufacturer instructions.

hiPSC-CM, -EC, -CF, and -CS drug treatment

For drug treatment experiments, day 30+ hiPSC-CMs, -ECs, and -CFs were re-seeded onto imaging-optimized black 96-well plates (ThermoFisher) at least 1 week before exposure. CSs were treated with drugs at 14 days after aggregation. Before treatment, SPEDOX-6 and UF DOX were either resuspended in RPMI 1640 medium (ThermoFisher) and B27 supplement with insulin (ThermoFisher) (hiPSC-CMs), EGM2 media (Lonza) containing 10 μM SB431542 transforming growth factor-beta inhibitor (Cayman Chemical), and 50 ng/mL vascular endothelial growth factor (Peprotech) (hiPSC-ECs), or EGM2 media containing 25 ng/mL fibroblast growth factor (Peprotech) (hiPSC-CFs). For 24, 48, and 72 h, each cell type was exposed to drug conditions in triplicate. hiPSC-CSs were also exposed to drug conditions in triplicate for 24, 48, and 72 h, but remained on clear U-bottom 96-well plates with 30-μL media collections taken daily.

hiPSC-CM/EC/CF/CS cytotoxicity assays

During drug treatment, 60-μL media collections were taken daily per triplicate in each drug condition to assess cell viability by a LDH assay via CyQUANT LDH Cytotoxicity Assay kit (ThermoFisher). Similarly, the absorbance-based CCK8 cytotoxicity/cell metabolism assay (Dojindo) was performed according to manufacturer instructions.

hiPSC-CM/CS functional assessments

hiPSC-CMs and hiPSC-CSs were incubated at 37°C and 5% CO_2 during contractility and calcium signaling assessments. hiPSC-CM contractions were counted via visual assessment to determine beats per minute. Fluorescent and brightfield videos from cells were taken at 15 frames per second over 10–20 s for each region of interest. Calcium transient signal is calculated automatically using a built-in ImageJ plugin, “Time Series Analysis.” The recorded signal is then preprocessed with detrending using a linear trendline filter in MATLAB R2022 to correct the baseline. For contraction profiles, signal analysis was conducted using a digital image correlation analysis (DIC). Contraction signal amplitude, full width at half maximum, and beat rates were extracted from the DIC’s outcome. For single sarcomere contractility assessment, ACTN2-GFP hiPSC-CMs were treated with selected drugs and video imaged at 100× magnification. Videos were analyzed in MATLAB R2022 using SarcTrack sarcomere analysis software, as shown previously

(Toepfer et al., 2019). Field potential measurements were taken from hiPSC-CMs plated into a multielectrode array (Axion) at 24, 48, and 72 h after initial drug addition. For calcium imaging assessment, WTC-GCaMP hiPSC-CMs were treated with selected drugs and video imaged at 20× magnification to be analyzed in MATLAB (Lu et al., 2015). hiPSC-CSs were video imaged at 10× and 20× after drug addition at 24, 48, and 72 h to assess contractility displacement via the VW-9000 Motion Analyzer on a BZ-X810 microscope (Keyence).

Statistical analysis

Data are presented as mean ± SE for a minimum of three independent experiments, unless indicated. Statistical significance was determined by repeated measurement for comparisons at multiple time points (Prism 9, GraphPad Software). For determining statistical significance between two groups, a value of $p < 0.05$ by Student *t* test was considered significant, represented by an asterisk (*). Three or more groups were analyzed by one-way ANOVA followed by Tukey's post hoc tests, and a value of $p < 0.05$ was considered significant, represented by an asterisk (*). NS, non-significance. Experiments were scored blind to treatment where possible.

SUPPLEMENTAL INFORMATION

Supplemental information can be found online at https://doi.org/10.1016/j.stemcr.2023.08.005.

AUTHOR CONTRIBUTIONS

M.A. and B.G., experimental execution, data collection, data analysis, and manuscript writing; M.M., S.P., Q.L., F.H., R.B.C., C.Y., Y.Z., X.F., A.E.G., A.J., and P.W.B., methodology, data collection, and data analysis; X.C. and A.S., conception and design, financial support, manuscript writing, and final approval of manuscript.

ACKNOWLEDGMENTS

Funding provided by the American Heart Association Career Development Award 856987 (to A.S.); the National Institutes of Health grants 2R01CA151610 and R21CA280458, Department of Defense grant W81XWH-18-1-0067, Uretsky BRCA Research Fund and Samuel Oschin Cancer Institute Research Development Fund (to X.C.); the Cedars-Sinai Cancer Center fund (to A.S and X.C.); and the Fashion Footwear Charitable Foundation of New York, Inc., the Margie and Robert E. Petersen Foundation, and Linda and Jim Lippman Fund (to A.E.G.). A.S. is supported by the Board of Governors Regenerative Medicine Institute at Cedars-Sinai, an In-Space Production Award (InSPA) from NASA, and the Donna and Jesse Garber Award for Cancer Research. A.S. and S.P. are supported by a California Institute for Regenerative Medicine (CIRM) Bridges Award. The ACTN2-GFP and Tubulin-RFP fluorescent reporter hiPSC lines were purchased from Coriell and the Allen Institute for Cell Science. The WTC-GCaMP hiPSC line was a kind gift from Dr. Bruce Conklin (UCSF) and Dr. Nathaniel Huebsch (Washington University in St. Louis). We thank the Cedars-Sinai Center for Bioinformatics and Functional Genomics core facility for their assistance with RNA-sequencing.

CONFLICT OF INTERESTS

C.Y. is a named inventor for patent applications regarding "Single Protein-Encapsulated Pharmaceutics for Enhancing Therapeutic Effects" and a shareholder of Sunstate Biosciences, LLC. The remaining authors have nothing to disclose.

Received: May 28, 2022
Revised: August 6, 2023
Accepted: August 7, 2023
Published: August 31, 2023

REFERENCES

Arzt, M., Pohlman, S., Mozneb, M., and Sharma, A. (2023). Chemically Defined Production of Tri-Lineage Human iPSC-Derived Cardiac Spheroids. Curr Protoc *3*, e767.

Asnani, A., Moslehi, J.J., Adhikari, B.B., Baik, A.H., Beyer, A.M., Boer, R.A.d., Ghigo, A., Grumbach, I.M., Jain, S., and Zhu, H. (2021). Preclinical Models of Cancer Therapy–Associated Cardiovascular Toxicity: A Scientific Statement From the American Heart Association. Circ. Res. *129*, e21–e34.

Burridge, P.W., Li, Y.F., Matsa, E., Wu, H., Ong, S.G., Sharma, A., Holmström, A., Chang, A.C., Coronado, M.J., Ebert, A.D., et al. (2016). Human induced pluripotent stem cell-derived cardiomyocytes recapitulate the predilection of breast cancer patients to doxorubicin-induced cardiotoxicity. Nat. Med. *22*, 547–556.

Giacomelli, E., Bellin, M., Sala, L., van Meer, B.J., Tertoolen, L.G., Orlova, V.V., and Mummery, C.L. (2017). Three-dimensional cardiac microtissues composed of cardiomyocytes and endothelial cells co-differentiated from human pluripotent stem cells. Development *144*, 1008–1017.

Giacomelli, E., Meraviglia, V., Campostrini, G., Cochrane, A., Cao, X., van Helden, R.W.J., Krotenberg Garcia, A., Mircea, M., Kostidis, S., Davis, R.P., et al. (2020). Human-iPSC-Derived Cardiac Stromal Cells Enhance Maturation in 3D Cardiac Microtissues and Reveal Non-cardiomyocyte Contributions to Heart Disease. Cell Stem Cell *26*, 862–879.e11.

Huebsch, N., Loskill, P., Mandegar, M.A., Marks, N.C., Sheehan, A.S., Ma, Z., Mathur, A., Nguyen, T.N., Yoo, J.C., Judge, L.M., et al. (2015). Automated Video-Based Analysis of Contractility and Calcium Flux in Human-Induced Pluripotent Stem Cell-Derived Cardiomyocytes Cultured over Different Spatial Scales. Tissue Eng. C Methods *21*, 467–479.

Kim, Y., Seidman, J.G., and Seidman, C.E. (2022). Genetics of cancer therapy-associated cardiotoxicity. J. Mol. Cell. Cardiol. *167*, 85–91.

Knowles, D.A., Burrows, C.K., Blischak, J.D., Patterson, K.M., Serie, D.J., Norton, N., Ober, C., Pritchard, J.K., and Gilad, Y. (2018). Determining the genetic basis of anthracycline-cardiotoxicity by molecular response QTL mapping in induced cardiomyocytes. Elife *7*, e33480.

Kratz, F. (2008). Albumin as a drug carrier: design of prodrugs, drug conjugates and nanoparticles. J. Contr. Release *132*, 171–183.

Litviňuková, M., Talavera-López, C., Maatz, H., Reichart, D., Worth, C.L., Lindberg, E.L., Kanda, M., Polanski, K., Heinig, M.,

Lee, M., et al. (2020). Cells of the adult human heart. Nature *588*, 466–472.

Lu, H.R., Whittaker, R., Price, J.H., Vega, R., Pfeiffer, E.R., Cerignoli, F., Towart, R., and Gallacher, D.J. (2015). High Throughput Measurement of Ca++ Dynamics in Human Stem Cell-Derived Cardiomyocytes by Kinetic Image Cytometery: A Cardiac Risk Assessment Characterization Using a Large Panel of Cardioactive and Inactive Compounds. Toxicol. Sci. *148*, 503–516.

Magdy, T., Jiang, Z., Jouni, M., Fonoudi, H., Lyra-Leite, D., Jung, G., Romero-Tejeda, M., Kuo, H.H., Fetterman, K.A., Gharib, M., et al. (2021). RARG variant predictive of doxorubicin-induced cardiotoxicity identifies a cardioprotective therapy. Cell Stem Cell *28*, 2076–2089.e7.

Magdy, T., Jouni, M., Kuo, H.H., Weddle, C.J., Lyra-Leite, D., Fonoudi, H., Romero-Tejeda, M., Gharib, M., Javed, H., Fajardo, G., et al. (2022). Identification of Drug Transporter Genomic Variants and Inhibitors That Protect Against Doxorubicin-Induced Cardiotoxicity. Circulation *145*, 279–294.

Merlot, A.M., Kalinowski, D.S., and Richardson, D.R. (2014). Unraveling the mysteries of serum albumin-more than just a serum protein. Front. Physiol. *5*, 299.

Moslehi, J.J. (2016). Cardiovascular Toxic Effects of Targeted Cancer Therapies. N. Engl. J. Med. *375*, 1457–1467.

Qiao, X., van der Zanden, S.Y., Wander, D.P.A., Borràs, D.M., Song, J.Y., Li, X., van Duikeren, S., van Gils, N., Rutten, A., van Herwaarden, T., et al. (2020). Uncoupling DNA damage from chromatin damage to detoxify doxorubicin. Proc. Natl. Acad. Sci. USA *117*, 15182–15192.

Rhee, J.W., Ky, B., Armenian, S.H., Yancy, C.W., and Wu, J.C. (2020). Primer on Biomarker Discovery in Cardio-Oncology: Application of Omics Technologies. JACC. CardioOncol. *2*, 379–384.

Sala, L., van Meer, B.J., Tertoolen, L.G.J., Bakkers, J., Bellin, M., Davis, R.P., Denning, C., Dieben, M.A.E., Eschenhagen, T., Giacomelli, E., et al. (2018). MUSCLEMOTION: A Versatile Open Software Tool to Quantify Cardiomyocyte and Cardiac Muscle Contraction In Vitro and In Vivo. Circ. Res. *122*, e5–e16.

Sayed, N., Ameen, M., and Wu, J.C. (2019). Personalized medicine in cardio-oncology: the role of induced pluripotent stem cell. Cardiovasc. Res. *115*, 949–959.

Sharma, A., Burridge, P.W., McKeithan, W.L., Serrano, R., Shukla, P., Sayed, N., Churko, J.M., Kitani, T., Wu, H., Holmström, A., et al. (2017). High-throughput screening of tyrosine kinase inhibitor cardiotoxicity with human induced pluripotent stem cells. Sci. Transl. Med. *9*, eaaf2584.

Toepfer, C.N., Sharma, A., Cicconet, M., Garfinkel, A.C., Mücke, M., Neyazi, M., Willcox, J.A.L., Agarwal, R., Schmid, M., Rao, J., et al. (2019). SarcTrack: An Adaptable Software Tool for Efficient Large-Scale Analysis of Sarcomere Function in hiPSC-Cardiomyocytes. Circ. Res. *124*, 1172–1183.

Yu, C., Huang, F., Chow, W.A., Cook-Wiens, G., and Cui, X. (2020). Single Protein Encapsulated Doxorubicin as an Efficacious Anticancer Therapeutic. Adv. Ther. *3*, 2000135.

Stem Cell Reports, Volume *18*

Supplemental Information

Protein-encapsulated doxorubicin reduces cardiotoxicity in hiPSC-cardiomyocytes and cardiac spheroids while maintaining anticancer efficacy

Madelyn Arzt, Bowen Gao, Maedeh Mozneb, Stephany Pohlman, Romina B. Cejas, Qizhi Liu, Faqing Huang, Changjun Yu, Yi Zhang, Xuemo Fan, Amelia Jenkins, Armando E. Giuliano, Paul W. Burridge, Xiaojiang Cui, and Arun Sharma

SUPPLEMENTAL FIGURES AND LEGENDS

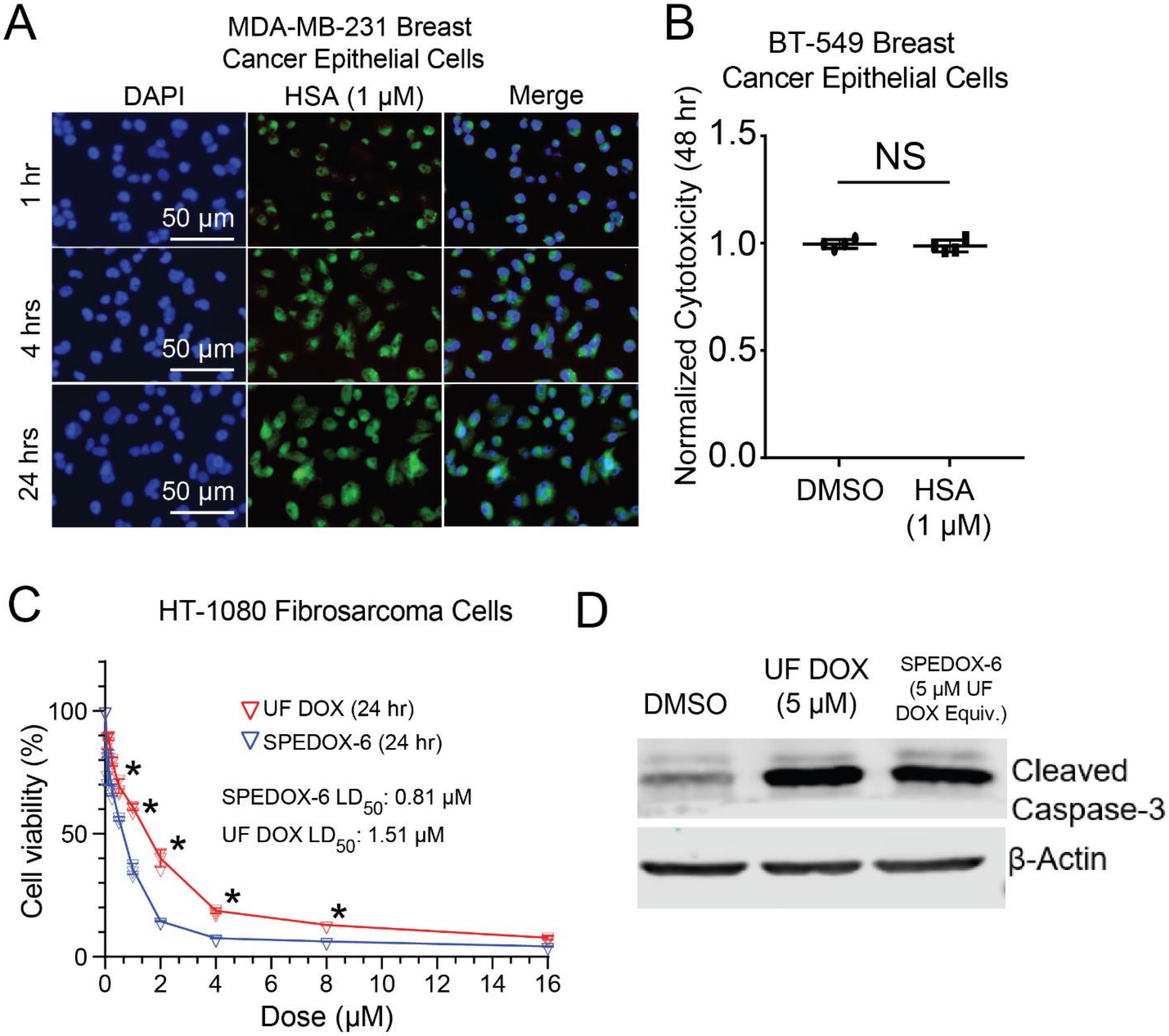

Figure S1 (related to Figure 1): Evaluation of SPEDOX-6 cytotoxicity in multiple human cancer cell lines. (A) Immunofluorescence staining following treatment of MDA-MB-231 human breast cancer cells with human serum albumin (HSA). HSA exhibits rapid nuclear accumulation in MDA-MB-231 cells within hours of treatment. **(B)** BT-549 breast cancer epithelial cells treated with either DMSO or HSA for 48 hours show no significant (NS) difference in cytotoxicity. * indicates $p<0.05$ by Student's t test. NS indicates non-significance. N=4 independent experiments. **(C)** Cell viability dose response assay conducted with unformulated doxorubicin (UF DOX) and SPEDOX-6 on HT-1080 fibrosarcoma cells for 24 hours. * indicates $p<0.05$ by Student's t test. N=3 independent experiments. **(D)** Western blot assay examining the protein expression of cleaved caspase-3 (apoptosis marker) in HT-1080 fibrosarcoma cells after UF DOX or SPEDOX-6 treatment.

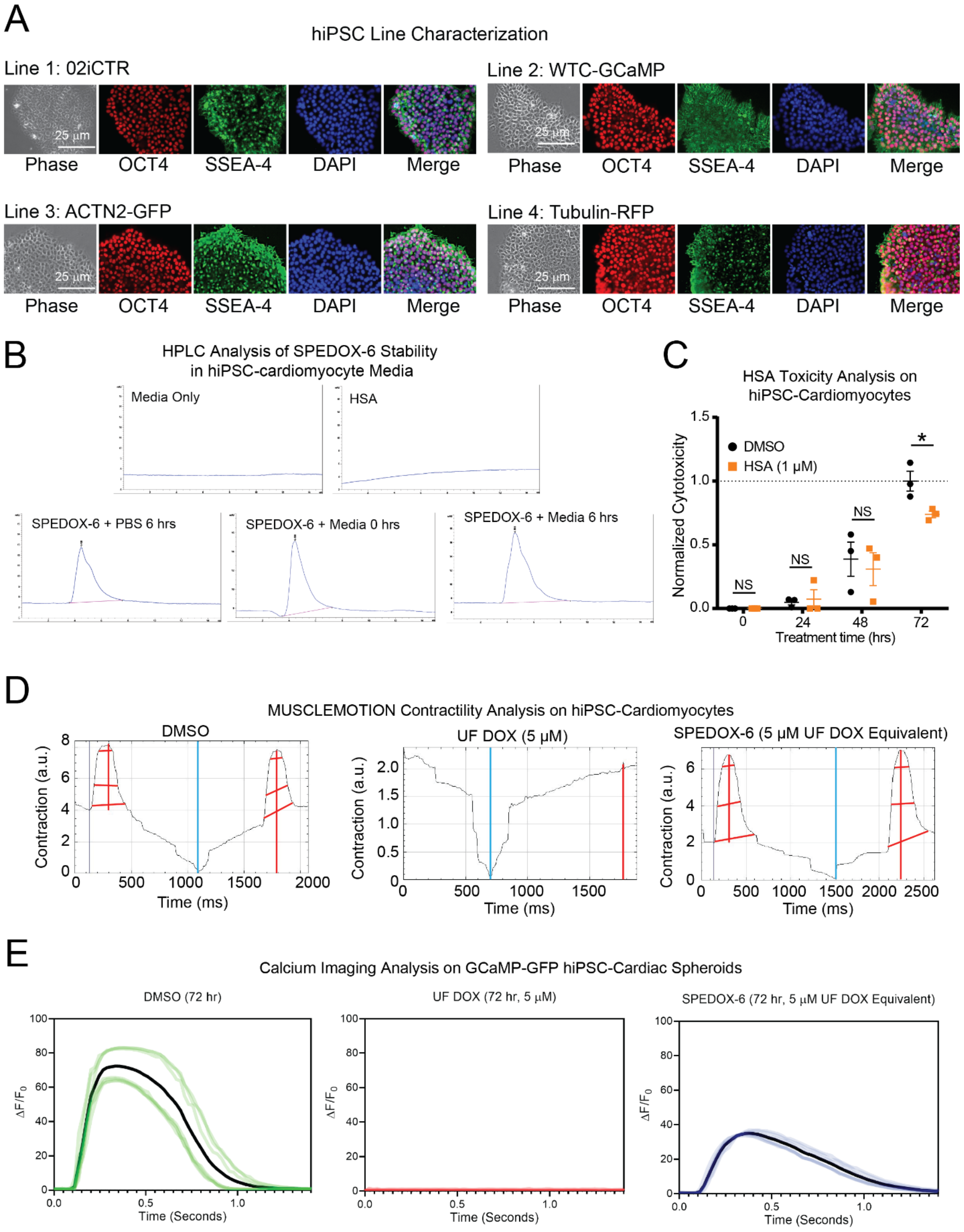
A
hiPSC Line Characterization
Line 1: 02iCTR
Line 2: WTC-GCaMP
Line 3: ACTN2-GFP
Line 4: Tubulin-RFP
25 µm
Phase
OCT4
SSEA-4
DAPI
Merge
B
HPLC Analysis of SPEDOX-6 Stability in hiPSC-cardiomyocyte Media
Media Only
HSA
SPEDOX-6 + PBS 6 hrs
SPEDOX-6 + Media 0 hrs
SPEDOX-6 + Media 6 hrs
C
HSA Toxicity Analysis on hiPSC-Cardiomyocytes
DMSO
HSA (1 µM)
Normalized Cytotoxicity
Treatment time (hrs)
NS
*
D
MUSCLEMOTION Contractility Analysis on hiPSC-Cardiomyocytes
DMSO
UF DOX (5 µM)
SPEDOX-6 (5 µM UF DOX Equivalent)
Contraction (a.u.)
Time (ms)
E
Calcium Imaging Analysis on GCaMP-GFP hiPSC-Cardiac Spheroids
DMSO (72 hr)
UF DOX (72 hr, 5 µM)
SPEDOX-6 (72 hr, 5 µM UF DOX Equivalent)
ΔF/F0
Time (Seconds)

Figure S2 (related to Figure 2, 3, 4): Characterization of hiPSC morphology and subsequent evaluation of differentiated hiPSC-cardiomyocyte HSA uptake and SPEDOX-6-induced toxicity (A) Immunofluorescence staining of hiPSC lines used in this project for protein markers of pluripotent stem cells, including OCT4 and SSEA-4. These markers indicate a high level of pluripotency gene expression in undifferentiated hiPSCs, a reflection of their overall health as pluripotent stem cells. These hiPSCs are then used for differentiations into hiPSC-derived cardiomyocytes (hiPSC-CMs), cardiac fibroblasts (hiPSC-CFs), and endothelial cells (hiPSC-ECs). **(B)** High performance liquid chromatography reveals SPEDOX-6 stability in cardiomyocyte culture medium. High performance liquid chromatography (HPLC) using a size exclusion Protein-Pak 125 Column (Waters, MAQ, 7.8 mm x 300 mm) column. The analytes were monitored at 480 nm. Large molecules, such as proteins, were eluted first, whereas small molecules were retained for a more extended period of time before elution. Chromatography results with media only, human serum albumin (HSA), SPEDOX-6, SPEDOX-6 added to media for 0 hours, or SPEDOX-6 added to media for 6 hours. Results indicate that 6 hours of mixing cardiomyocyte cell culture medium with SPEDOX-6 did not alter the absorption profile, suggesting that SPEDOX-6 stability is not substantially affected by the culture medium. Y-axis: absorption at 480 nm, maximum absorption wavelength of doxorubicin. **(C)** Human serum albumin (HSA) treatment alone in hiPSC-cardiomyocytes (hiPSC-CMs) does not enhance cytotoxicity above DMSO. hiPSC-CMs treated with HSA show no enhancement in cytotoxicity above DMSO via lactate dehydrogenase (LDH) assay. N=3 independent experiments. * indicates $p<0.05$ by Student's t test. **(D)** Automated analysis of 2D hiPSC-CM contractility after drug treatment using MUSCLEMOTION software shows improvement in contractility in SPEDOX-6 treatment compared to unformulated doxorubicin (UF DOX). Videos of hiPSC-cardiomyocyte (hiPSC-CM) contractility after (A) DMSO, (B) UF DOX, or (C) SPEDOX-6 treatment were analyzed using MUSCLEMOTION open-source software. This tool is a plugin for ImageJ that enables automated measurement of cardiomyocyte movement from input video files. Blue lines indicate the reference frame used for cell displacement measurements (Y-axis) over time (X-axis). Red lines highlight automatically detected cellular contractions. **(E)** Analysis of calcium cycling after drug treatment in hiPSC-cardiac spheroids (hiPSC-CSs) shows improvement in calcium handling in SPEDOX-6 treatment compared to unformulated doxorubicin (UF DOX). Videos were taken of hiPSC-CSs containing WTC-GCaMP calcium reporter hiPSC-CMs after (A) DMSO, (B) UF DOX, or (C) SPEDOX-6 treatment. See Supplemental Video 4.

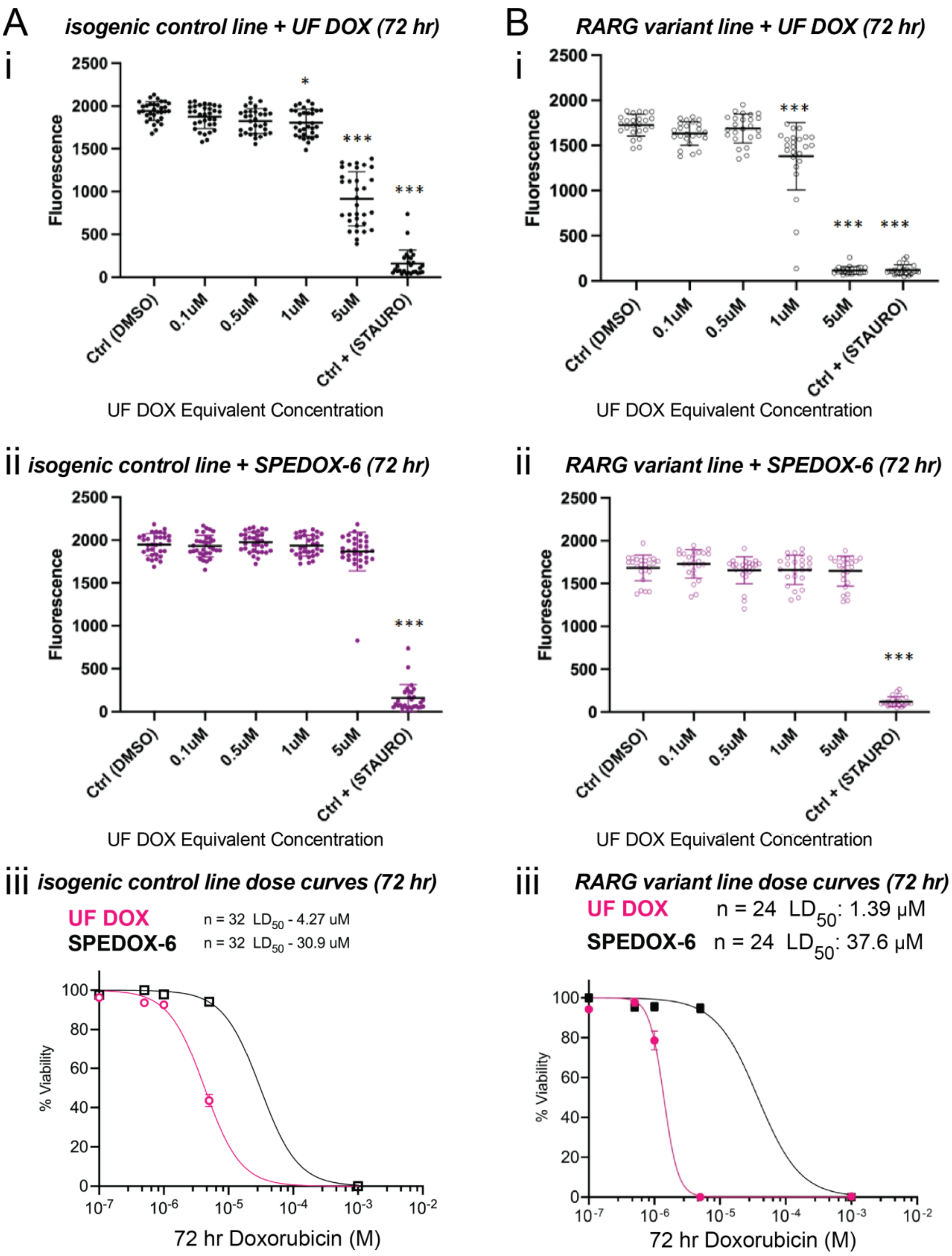

A
i
isogenic control line + UF DOX (72 hr)
Fluorescence
Ctrl (DMSO)
0.1uM
0.5uM
1uM
5uM
Ctrl + (STAURO)
UF DOX Equivalent Concentration
B
i
RARG variant line + UF DOX (72 hr)
Fluorescence
UF DOX Equivalent Concentration
ii
isogenic control line + SPEDOX-6 (72 hr)
Fluorescence
UF DOX Equivalent Concentration
ii
RARG variant line + SPEDOX-6 (72 hr)
Fluorescence
UF DOX Equivalent Concentration
iii
isogenic control line dose curves (72 hr)
UF DOX
n = 32 LD50 - 4.27 uM
SPEDOX-6
n = 32 LD50 - 30.9 uM
% Viability
72 hr Doxorubicin (M)
iii
RARG variant line dose curves (72 hr)
UF DOX
n = 24 LD50: 1.39 μM
SPEDOX-6
n = 24 LD50: 37.6 μM
% Viability
72 hr Doxorubicin (M)

Figure S3 (related to Figure 2): SPEDOX-6 induces less cytotoxicity than unformulated doxorubicin (UF DOX) in isogenic and patient-specific hiPSC-cardiomyocytes (hiPSC-CMs) harboring single nucleotide polymorphism (rs2229774) in retinoic acid receptor-γ (RARG). Reazurin blue fluorescence-based cell viability dose response assay with UF DOX or SPEDOX-6 treatment for 72 hours in **(A)** an isogenic control hiPSC-CM line or **(B)** hiPSC-cardiomyocytes (hiPSC-CMs) harboring single nucleotide polymorphism (rs2229774) in retinoic acid receptor-γ (RARG). DMSO serves as negative control for cytotoxicity and stauro-sporine (STAURO) serves as positive control for cytotoxicity. UF DOX treatments shown in subpanels i and SPEDOX-6 treatments shown in subpanels ii. Subpanels iii show cell viability dose response curves for UF DOX or SPEDOX-6 drug treatment in either isogenic or RARG mutant hiPSC-CMs. * indicates $p<0.05$, ** indicates $p<0.01$, and *** indicates $p<0.001$ by Student's t test between DMSO and indicated condition. LD_{50} indicates drug concentration at which 50% loss in cell viability occurs.

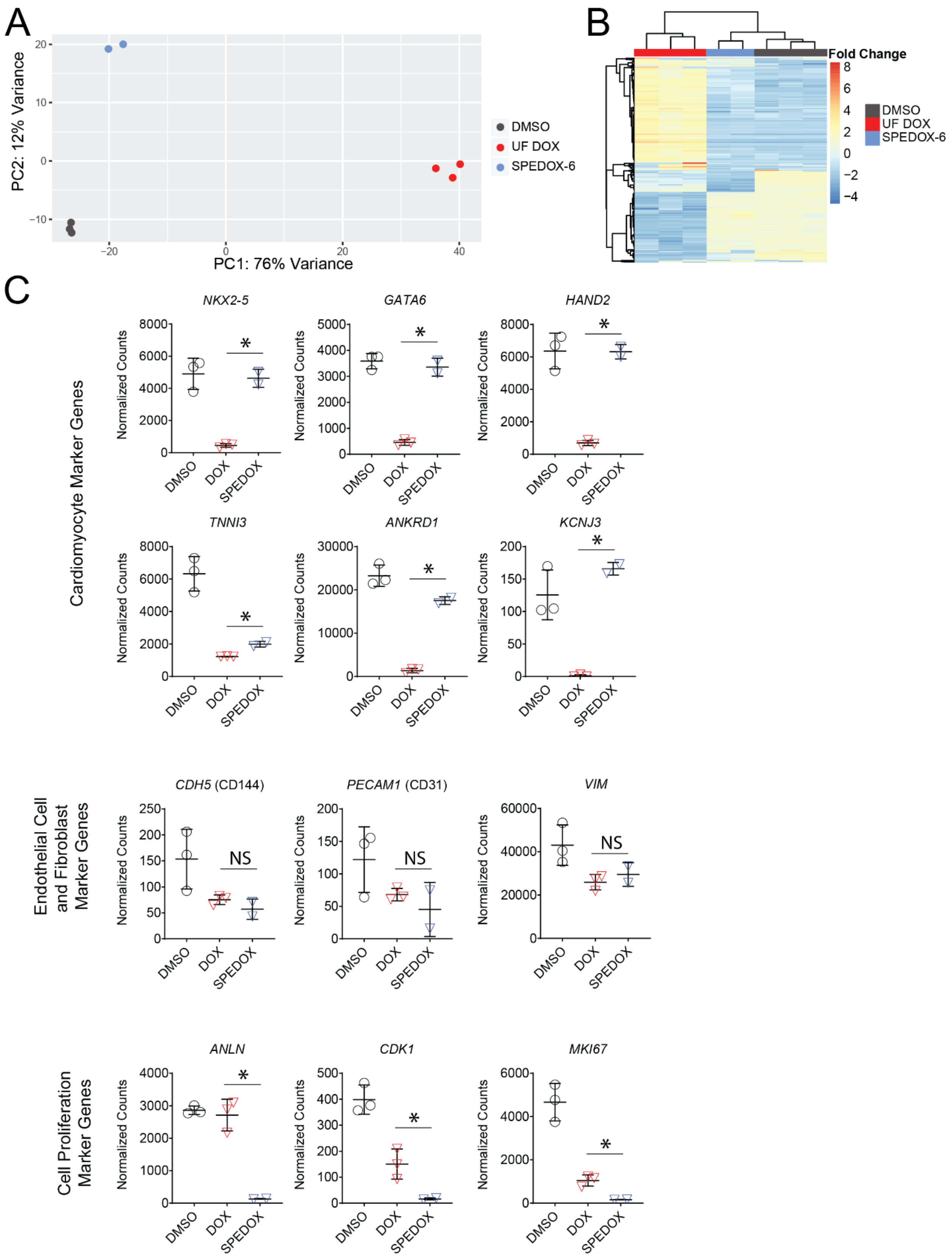

A
PC2: 12% Variance
PC1: 76% Variance
DMSO
UF DOX
SPEDOX-6
B
Fold Change
DMSO
UF DOX
SPEDOX-6
C
Cardiomyocyte Marker Genes
NKX2-5
GATA6
HAND2
TNNI3
ANKRD1
KCNJ3
Endothelial Cell and Fibroblast Marker Genes
CDH5 (CD144)
PECAM1 (CD31)
VIM
Cell Proliferation Marker Genes
ANLN
CDK1
MKI67
Normalized Counts
DMSO
DOX
SPEDOX
NS

Figure S4 (related to Figure 4): Bulk RNA-sequencing analysis after drug treatment in hiPSC-cardiac spheroids (hiPSC-CSs) indicates cell type-specific transcriptional responses in SPEDOX-6 treatment compared to unformulated doxorubicin (UF DOX). (A) Principal component analysis (PCA) plot of drug-treated cardiac spheroid RNA-seq samples. Treatment was for 48 hours. UF DOX added at 5 µM and SPEDOX-6 added at 5 µM UF DOX equivalent dose. N=3 independent experiments for DMSO and UF DOX. N=2 independent experiments for SPEDOX-6. **(B)** Heatmap of drug-treated cardiac spheroid samples indicating clustering by condition. **(C)** Representative genes indicating the cell type-specific impact of drugs on hiPSC-CMs (*NKX2-5, GATA6, HAND2, TNNI3, ANKRD1, KCNJ3*), hiPSC-ECs (*CDH5, PECAM1*), and hiPSC-CFs (*VIM*). Other genes of interest include proliferation markers *ANLN, CDK1* and *MKI67*. * indicates $p<0.05$ determined by one-way ANOVA with Tukey's post hoc test. NS indicates not significant.

SUPPLEMENTAL EXPERIMENTAL PROCEDURES

hiPSC Culture. All hiPSC cultures were maintained at 37°C in a Forma Steri-Cycle CO_2 incubator (ThermoFisher) with 5% CO_2. Cells were routinely maintained in mTeSR medium (STEMCELL Technologies) on 100 µg/mL Matrigel (Corning) and passaged every 3-4 days using 0.5 mM EDTA in PBS. hiPSCs were replenished daily with 2 mL mTeSR medium and routinely tested for mycoplasma using a MycoAlert Plus Kit (Lonza).

hiPSC-CM Differentiation. Healthy control hiPSCs derived in the Cedars-Sinai Regenerative Medicine Institute were differentiated into hiPSC-CMs following a chemically defined protocol with slight variations in the duration and concentration of CHIR99021 GSK3-beta inhibitor (Cayman Chemical) treatment, as described previously (Sharma et al., 2018). hiPSC-CMs were dissociated with TrypLE Select Enzyme 10X (ThermoFisher) when re-plated and re-seeded on Matrigel-coated plates.

hiPSC-EC Differentiation. hiPSC-ECs were produced following a chemically defined protocol with modulation of CHIR99021 GSK3-beta inhibitor as described previously (Liu et al., 2018). At day 12 of differentiation, ECs were sorted using magnetic activated cell sorting (MACS) and a CD144 antibody (Miltenyi Biotec). $CD144^+$ cells were then cultured and expanded with EGM2 media changes every 2 days.

hiPSC-CF Differentiation. hiPSC-CFs were produced following a chemically defined protocol with variation of CHIR99021 GSK3-beta inhibitor. Concentrations of CHIR99021 ranging from 8-12 µM were added to RPMI 1640 and B27 minus insulin medium for days 0-2 on hiPSCs. From days 2-3, the medium was substituted to just RPMI 1640 and B27 minus insulin (ThermoFisher), with the addition of 2 µM Wnt-C59 (ThermoFisher) at day 3 for days 3-5. EGM2 media containing 25 ng/mL FGF was then added and changed every 2 days until day 12.

hiPSC-CS Differentiation. hiPSC-CMs, hiPSC-ECs, and hiPSC-CFs were either thawed or re-plated, counted, and seeded together onto clear U-bottom 96-well plates in an 8:1:1 ratio of CMs, ECs, and CFs, respectively, for a total of 20,000 cells, as described previously (Arzt et al., 2023). A multi-lineage (hiPSC-CM, hiPSC-EC, and hiPSC-CF) medium consisting of RPMI 1640, 505 µg/mL recombinant human albumin (ScienCell Research Laboratories), 220 µg/mL L-ascorbic acid 2-phosphate (Fujifilm Wako Chemicals), 20 µg/mL insulin (ThermoFisher), 20% fetal bovine serum (ThermoFisher), and small molecules from the EGM2 media kit (Lonza) was used for seeding and changed every 2 days for 1-2 weeks for the spheroids to form and start contracting.

Immunofluorescence. Immunofluorescence was conducted according to published protocols (Sharma et al., 2017). Briefly, the hiPSCs, hiPSC-CMs, hiPSC-ECs, and hiPSC-CFs were fixed with 4% PFA and stained with selected antibodies at 1:200 dilution (Origene, Abcam). Cells were washed with phosphate buffer saline (PBS) and then incubated for 2 hours at room temperature in the dark with Alexa-Fluor-conjugated secondary antibodies (1:500, diluted in 3% BSA, ThermoFisher). Wells were washed again with PBS and incubated for 10 minutes at room temperature in the dark with DAPI (10% diluted in PBS, ThermoFisher) and imaged with an EVOS FL microscope (ThermoFisher) or BZ-X810 microscope.

Transcriptional Analysis of DMSO, UF DOX, and SPEDOX-6-treated hiPSC-CSs. Total RNA was isolated from hiPSC-CSs treated with DMSO, UF DOX (5 µM), and SPEDOX-6 (5 µM UF DOX equivalent) using a RNeasy Mini Kit (QIAGEN) with on-column DNase digestion. Nine spheroids were treated per condition for 48 hours at 40,000 cells per spheroid, following previously published ratios of hiPSC-CMs, hiPSC-ECs, and hiPSC-CFs (Arzt et al., 2023). Each spheroid was washed with 200 µL PBS and then followed by a wash with 350 µL Buffer RLT containing β-mercaptoethanol and DTT to lyse the cells. Still in Buffer RLT, 3 spheroids from 1 condition were then transferred to an Eppendorf tube for a single sample replica. 3 replicates of 3 spheroids per condition was generated, or 120,000 cells per replicate. The contents of each tube were pipetted up and down and centrifuged at 8000 x g for 3 minutes. The supernatant was then collected for further processing. 350 µL 70% ethanol was added per tube and the contents were transferred to RNeasy MinElute spin columns inside 2 mL collection tubes which were centrifuged for 15 seconds at 8000 x g. Buffer RW1 was then added to each column at 350 µL and spun for 15 seconds at 8000 x g. A mixture of Buffer RDD and DNase 1 was then added to each column at 80 µL and incubated

at room temperature for 15 minutes. An additional 350 μL Buffer RW1 was added to each collection tube and centrifuged at 8000 x g for 15 seconds. A final addition of 500 μL 80% ethanol was added to each column and centrifuged at 8000 x g for 2 minutes. After transferring the spin column to a new collection tube, each column was spun at 5 minutes at full speed. The columns were transferred to a 1.5 mL tube and 14 μL RNase free water was added to the center of each column followed by centrifugation at full speed for 1 minute to elute the RNA. After a verification of the RNA yield of each tube via a NanoDrop 2000 Spectrophotometer (ThermoFisher), the samples were submitted to the Cedars-Sinai Genomics Core for ultra-low input total RNA sequencing. RNA integrity was checked with an Agilent 2100 Bioanalyzer and only samples with an RNA integrity (RIN) score >9 were selected for cDNA library construction. Libraries were prepared with SMART-Seq v4 Ultra Low Input RNA Kit (Takara) and sequenced on a NextSeq 500 (Illumina) using single-end 75 bp reads at the Cedars-Sinai Genomics Core. Demultiplexing and conversion of raw sequencing data to FASTQ was performed with Illumina bcl2fastq software. FastQC was used to assess sequence quality and reads were aligned to the GRCh38 genome assembly (GENCODE 28) using Salmon version 0.11.3. Gene-level read counts were quantified with Salmon, and differential gene expression and normalization was subsequently performed using DESeq2. Data from this project is available at Gene Expression Omnibus (GEO) under accession number GSE235470.

SUPPLEMENTAL REFERENCES

Arzt, M., Pohlman, S., Mozneb, M., and Sharma, A. (2023). Chemically Defined Production of Tri-Lineage Human iPSC-Derived Cardiac Spheroids. Current protocols *3*, e767.

Liu, C., Cheng, L., Chen, C.L., and Sayed, N. (2018). Generation of Endothelial Cells from Human Induced Pluripotent Stem Cells. Bio-protocol *8*, e3086.

Sharma, A., Burridge, P.W., McKeithan, W.L., Serrano, R., Shukla, P., Sayed, N., Churko, J.M., Kitani, T., Wu, H., Holmstrom, A., *et al.* (2017). High-throughput screening of tyrosine kinase inhibitor cardiotoxicity with human induced pluripotent stem cells. Science translational medicine *9*.

Sharma, A., Toepfer, C.N., Ward, T., Wasson, L., Agarwal, R., Conner, D.A., Hu, J.H., and Seidman, C.E. (2018). CRISPR/Cas9-Mediated Fluorescent Tagging of Endogenous Proteins in Human Pluripotent Stem Cells. Current protocols in human genetics *96*, 21 11 21-21 11 20.
